# To evaluate the impact of opening up ownership of pharmacies in South Africa

**DOI:** 10.1186/s40545-020-00232-4

**Published:** 2020-08-07

**Authors:** Rajatheran Moodley, Fatima Suleman

**Affiliations:** Discipline of Pharmaceutical Sciences, School of Health Sciences, Westville Campus, University of KwaZulu-Natal, Private Bag X54001, Durban, KZN 4000 South Africa

**Keywords:** Ownership, South Africa, Liberalisation, Medicine access, Pharmacy, Ownership

## Abstract

**Background:**

Following the democratic elections in 1994 the South African private pharmaceutical services were mostly in metropolitan centred with a scattering of pharmacies in less densely populated areas. The Government introduced regulations relating to the ownership and licensing of pharmacies on the 25th of April 2003 to improve access to pharmaceutical services by removing ownership restriction to only pharmacists.

**Objective:**

To assess the outcomes of the policy implementation in improving access to pharmacies.

**Method:**

The register of pharmacies at the South African Pharmacy Council was analysed from 1994 to 2014. Each registration was assigned GPS coordinates using Q-GIS(V3.6) and mapped per province at a district level, following clean-up and verification of the register. New registrations were also categorised as either corporate or independent pharmacy. Population census was obtained from Statistics South Africa and used to determine the number of pharmacies per 100,000 population.

**Main outcome measure(s):**

Number of active pharmacies; Number of independent pharmacies; number of pharmacies in each district.

**Results:**

The number of active pharmacies increased from 1624 at the end of 2003 to 3021 by 2014. The closure rate decreased from 137 to 86 pharmacies per year post regulations, a 37.23% reduction with a net gain of approximately 127 pharmacies per year. About 38.30% of all pre-2003 pharmacies (622 of 1624) closed by 2014. The population increase in the study period was approximately 20.66% but the overall growth of pharmacies was only 1.88 pharmacies per 100,000 population (3.55 to 5.43). Following the regulations in 2004, 23.9% of pharmacies active within the system closed between 2004 and 2014, of which, 91.7% of them were independent pharmacies.

**Conclusion:**

Opening up of pharmacy ownership in South Africa increased the number of pharmacies in the country but did not result in increased access in previously less populated areas. There was still clustering of pharmacies in a well resourced areas, with a steady growth in corporate pharmacy (35%) ownership.

## Impact of findings on practice statements

1. Opening up ownership of pharmacies to non-pharmacists may not result in a large increase in pharmacy access in previously disadvantaged and rural areas
2. Policymakers need to consider other incentives to improve access in underserved areas.
3. Policymakers should monitor implementation of the policy to avoid monopolies being developed

## Background

Following the 1994 democratic elections the new Government in South Africa had the opportunity to introduce policies that ensured the availability and accessibility of cost-effective medicines to all South Africans. A National Pharmaceutical Policy Committee was established by the Government post elections in April 1994 [1], which led to the publication of the National Drug Policy [2]. The key concept related to pharmacy ownership was contained in the following statement; “Where it is deemed to be in the interests of the public, and provided that comprehensive pharmaceutical care is ensured, ownership of pharmacies by laypersons and other health care professionals will be considered [2].”

It is important to reflect on the intention of the Minister in introducing the Bill to parliament in 1997 for debate. The Group Areas Act (1950) defined residential zones and confined healthcare professionals to their own ethnic communities [3]. Black pharmacists [4] who qualified in the 80’s and early 90’s were not allowed to own pharmacies in urban areas (defined as per the National Spatial Development Framework Draft 2018 as “Urban areas are characterised by large communities living at high residential densities, a variety of employment opportunities, and high-intensity business and commercial areas”) [5], where trade was lucrative and profitable. Private pharmaceutical services were only accessible to affluent communities situated in metropolitan areas [6]. A metropolitan area is defined as a large densely populated city classified as Category A municipalities described in section 155(1) of the Constitution and Municipal Structures Act (Act 117 of 1998) [7]. The Bill sought to improve access to pharmaceutical services by removing restriction of ownership to only pharmacists. Further debate centred around the Minister’s powers in determining who should own pharmacies, and ownership being determined on a need basis. Part of the motivation heard in parliament [4] was that opening up of ownership would reduce the price of medicines, promote healthy competition and create more jobs.

The Regulations Relating to the Ownership and Licensing of Pharmacies was published in Notice No. 553 of 25 April 2003 [8] where the responsibility to issue a license was moved from the South African Pharmacy Council to the National Department of Health. Unlike many low income countries where pharmacy oversight, regular inspection and law enforcement is weak [9], the South African Pharmacy Council has a well-defined and stringent process.

In most countries where deregulation was attempted, the rationale for change centred around the need for increased competition, containment of pharmaceutical expenditure, improved access to pharmaceutical care and opening of new outlets in areas of need [10]. The Österreichisches Bundesinstitut für Gesundheitswesen Austrian Health Institute (OBIG) 2006 report [10] of the European Union (EU) countries indicated that 17 of the 25 member nations operated restricted ownership of pharmacies. The study went further to do a comparative analysis of three EU countries that were regulated i.e. Austria, Finland and Spain compared to the deregulated states of Ireland, Netherlands and Norway. The study showed a strong increase in the number of pharmacies in the deregulated member states accompanied by urban clustering and fewer municipalities having access to service.

A 2015 survey conducted by the International Pharmaceutical Federation (FIP) [11] in 71 countries covering 80% of the world’s population indicated that 66% of pharmacy ownership is non-exclusive to pharmacists and the balance of 34% (24 countries) were exclusive. Non pharmacist ownership ranged from state ownership to complete liberalisation. Other factors that determined ownership related to workforce capacity where the number of pharmacists may not be sufficient to cover the areas of need. Some countries have liberalisation but provide additional restrictions [11], the most frequent being restricting other authorized non-pharmacist prescribers from ownership, banning vertical integration in a supply chain, or restricting horizontal integration to prevent dominance. Strong regulated environments are built on restricted ownership to pharmacists, combined with geographic conditions [12] based on number of inhabitants per pharmacy and minimum distance from each other. This is meant to create a spread of pharmacies across geographic areas allowing for sustainability.

Challenges of restrictive ownership in Germany and Italy were brought to the European Court of Justice [11]. The court ruled that restriction with the justification of safety and quality is allowed. Two other countries, Hungary (2009) and Estonia (2015) [13], returned to regulated ownership based on professional independence of the pharmacists, lack of rural improvement, and financial unviability of the remaining pharmacies. In Africa, some countries such as Chad, Senegal, and Cameroon restrict ownership to pharmacists while Kenya and Nigeria, follow the South African model of liberal ownership. Countries with pro-competitive policies driven by competition authorities often drive deregulation [14].

In countries where ownership is exclusive to pharmacists [11] there is an understanding that community pharmacists form an extension of the healthcare system and provide an essential public service. These models exist extensively in Africa, Eastern Mediterranean, Australia and Europe. Multiple models of open ownership and restricted ownership in the United States (US) exist as in the case of South and North Dakota respectively. A 1963 state law restricting ownership to pharmacists was tested via the North Dakota Pharmacy Ownership Initiative [15] in November 2014 where a chain pharmacy group attempted to have the law repealed and lost in a public referendum. It was shown that across every key measure of pharmaceutical care including prescription prices, levels of patient care and most importantly rural access, North Dakota outperformed other states [16].

Other models of ownership which include non-governmental organisations, charities, religious groups and humanitarian organisations [11] are found in 28% of countries surveyed in a study by the Federation of International Pharmacy (FIP). Brazil has a unique model of municipal owned community pharmacies (Farmacias Populares do Brasil) [11] dispensing medicines off their essential medicine lists and employing pharmacists. Since 2009 when Sweden liberalised pharmacy ownership the sector is dominated by chains and independents [13]. The rationale for the deregulation which included pricing, efficiency and usage of medicine were replaced by diversity, entrepreneurship and privatisation goals [13].

The aim of this research was to explore the impact of opening up of ownership on rural access and ownership type before and after the introduction of the regulation in South Africa. There is limited research in this area, especially from middle- and low-income countries. It is important to understand if policy objectives can be met, or if unintended consequences occur.

## Methods

Although licenses are granted by the National Department of Health since 2004, service can only be activated with a SAPC certificate of registration. Thus, an analysis of the South African Pharmacy Council registers for the period 1994 to 2014 was conducted. The register data was cleaned, and allocation was done in terms of provinces. A verification process involving reconciling register records with Medpages [17] followed by random telephone sampling was conducted. Community pharmacies were classified and mapped as independent and corporate, and compared to the pre-2004 data. Opening and closures of pharmacies through the study period was recorded. Dates of Opening of new pharmacies, transfer of ownership, and closures are listed on the register. This was used in the year on year adjustment. Based on the registered ownership in the Council database, pharmacies were classified in terms of listed companies and non-listed. All pharmacies in the non-listed category was assumed to be Independent.

Global Positioning System (GPS) coordinates were assigned using Q-GIS (V3.6) before mapping at a district level. Population census (2001) and 2016 Community Survey to determine pharmacies per 100,000 population was obtained from Statistics South Africa (StatsSA) [18], as the previous community survey was published in 2011 and thought to be too dated to use in this study. The entire population was used as the denominator as community pharmacy serves both the private insured and the public in general. Both district and municipal information was sourced from the Municipal Demarcation Board [19]. According to the Municipal Demarcation Board [20], all major spatial restructuring of municipal/district boundaries took place prior to the 5 December 2000 local elections. Thus, for the duration of the study (2003–2014) there was little to no restructuring. The deprivation index and quintile allocation calculation were done in 2013/14 based on the 2011 census (District Health Barometer (2016/17)) [21], and this classification was used in this study.

## Results

The number of active pharmacies (Table 1) increased from 1624 in 2003 to 3021 in 2014. The closure rate reduced from 137 per year pre 2004 to 86 per year post regulations, a 37.23% reduction, gaining 127 pharmacies per year. The net gain was largest in Gauteng (39.51%) with Eastern Cape (1.93%), Northern Cape (1.36%), Free State (5.08%), North West (8.02%) and Mpumalanga (7.30%) showing increases in the number of new pharmacies. Of the pharmacies that were open in 2004 (Pre-2003 pharmacies) 38,30% (622 of 1624) were closed by 2014.

**Table 1:** Summary of Community Pharmacy Availability and Ownership Type from Pre 2003 to 2014

		Free State	Gauteng	Kwa Zulu-Natal	Limpopo	Northern Cape	Western Cape	Eastern Cape	Mpumalanga	North West	Total
**Pre2004 Registered Pharmacies**	**Pre 2004 Registered**	217	1449	671	122	68	578	213	185	208	**3711**
	**Closed before 1994**	59	393	132	20	18	111	3	57	64	**857**
	**Closed 1995–2003**	85	529	224	45	18	189	8	49	83	**1230**
	**Active in 2004**	73	527	315	57	32	278	202	79	61	**1624**
	**Rate of Closure per year (1995–2003)**	9.44	58.78	24.89	5.00	2.00	21.00	0.89	5.44	9.22	**136.67**
	**Closed Post 2003**	22	210	108	18	10	113	115	17	9	**622**
	**Active in 2014**	51	317	207	39	22	165	87	62	52	**1002**
	**Pharmacy to 100,000 Population ratio**	2.78	5.55	3.30	1.14	3.25	6.01	2.88	2.35	1.99	**3.55**
**Post2004 Registered Pharmacies**	**Post 2003 Registered**	108	914	340	181	42	342	147	139	133	**2346**
	**Independent Pharmacy Closure**	14	131	43	14	13	37	4	19	9	**284**
	**Corporate Pharmacy Closure**	1	21	5	0	0	11	1	1	3	**43**
	**Rate of Closure per year (2004–2014)**	3.36	32.91	14.18	2.91	2.09	14.64	10.91	3.36	1.91	**86.27**
	**Independent Active 2014**	62	472	192	148	16	144	94	91	84	**1303**
	**Corporate Active 2014**	31	290	100	19	13	150	48	28	37	**716**
	**Total Active in 2014**	144	1079	499	206	51	459	229	181	173	**3021**
	**Net gain/year**	6.45	50.18	16.73	13.55	1.73	16.45	2.45	9.27	10.18	**127.00**
	**Percentage Net Gain**	5.08	39.51	13.17	10.67	1.36	12.96	1.93	7.30	8.02	**100.00**
	**Pharmacy/100000 Population ratio**	5.08	8.05	4.51	3.55	4.27	7.31	3.27	4.17	4.62	**5.43**
**Population Census**	**2001**	2,623,956	9,501,134	9,535,936	4,995,535	983,653	4,624,336	7,022,968	3,365,886	3,072,342	**45,725,746**
	**2016**	2,834,715	13,399,725	11,065,245	5,799,091	1,193,783	6,279,731	6,996,974	4,335,964	3,748,437	**55,653,665**

The census indicated a population growth of 20.66% but pharmacies grew by only 1.88 pharmacies per 100,000 population (3.55 to 5.43). Pharmacies have continued to close during the identified study period (2004–2014) as follows: 622 of the pre 2003 registrations, 43 corporate and 284 independents registered post 2003; 23.9% of active pharmacies closed between 2004 and 2014 of which 91.7% were independent pharmacies.

Most provinces show a similar percentage closure of new pharmacies (2004–2014) – Western Cape (14%), Gauteng (16.6%), KwaZulu-Natal (14.1%), Free State (13.9%), and Mpumalanga (14.4%). The more rural provinces such as the Eastern Cape (3.4%), North West (9.0%) and Limpopo (7.7%) showed a lower closure rate with the Northern Cape being most affected as 31% of new pharmacies closed within the study period.

From Table 2, it can be seen that Manguang district in the Free State showed a substantial increase in the number of pharmacies from 19 (2004) to 47 (2014). The majority are located in densely populated areas. Increases in all other districts remained low with the Xhariep district having only 5 pharmacies by 2014. Little or no improvement was seen in the sparsely populated rural settlements.

**Table 2:** Opening and Closing of Pharmacies at District Level

District	Deprivation		Population		Pharmacies Registered Pre 2003					Pharmacies Registered Post 2003	
	Quintile	Dep. Index	2001	2016	Inactive in 2014			Active in 2014	Total Active in 2003	Active in 2014	Inactive in 2014
					Total	Active in 2003	Inactive in 2003				
**Free State**											
**Thabo Mofutsanyane**	3	46,082	725,939	779,330	26	3	23	13	16	**15**	**2**
**Fezile Dabi**	4	29,952	460,315	494,777	24	4	20	11	15	**20**	**2**
**Lejweleputswe**	4	42,767	657,012	646,920	57	5	52	14	19	**19**	**2**
**Xhariep**	3	19,756	135,250	125,884	6	1	5	3	4	**2**	**0**
**Mangaung**	5	43,466	645,440	787,804	53	9	44	10	19	**37**	**9**
**Total**			**2,623,956**	**2,834,715**	**166**	**22**	**144**	**51**	**73**	**93**	**15**
**Kwa Zulu-Natal**											
**Umkhanyakudi**	1	26,390	573,341	689,091	7	0	7	3	3	7	1
**Zululand**	1	46,844	854,779	892,310	18	2	16	5	7	9	2
**Uthungulu/King Cetshwayo**	2	13,210	885,964	971,135	16	3	13	16	19	18	7
**Umzinyathi**	1	18,719	480,413	554,883	12	0	12	1	1	9	2
**Amajuba**	3	31,444	468,036	531,328	25	5	20	6	11	12	1
**Uthukela**	2	20,149	656,984	706,589	11	3	8	6	9	10	1
**Umgungundlovu**	3	46,784	927,845	1,095,865	63	24	39	23	47	42	4
**Illembe**	2	11,018	560,389	657,613	14	4	10	6	10	11	3
**Ethekwini**	5	35,431	3,090,121	3,702,231	271	61	210	122	183	151	26
**Harry Gwala**	1	24,167	334,033	510,864	5	1	4	3	4	7	0
**Ugu**	2	17,593	704,031	753,336	22	5	17	16	21	16	1
**Total**			**9,535,936**	**11,065,245**	**464**	**108**	**356**	**207**	**315**	**292**	**48**
**Mpumalanga**											
**Ehlanzeni**	3	26,696	1,447,052	1,754,931	29	4	25	12	16	55	10
**Gert Sibanda**	3	26,696	900,007	1,135,409	46	2	44	30	32	21	4
**Nkangala**	4	27,061	1,018,827	1,445,624	48	11	37	20	31	43	6
**Total**			**3,365,886**	**4,335,964**	**123**	**17**	**106**	**62**	**79**	**119**	**20**
**Limpopo**											
**Mopani**	2	22,341	1,061,448	1,159,186	16	3	13	5	8	42	3
**Vhembe**	2	13,210	1,198,055	1,393,949	7	4	3	6	10	33	2
**Capricorn**	2	16,497	1,154,691	1,330,436	33	10	23	8	18	45	5
**Waterberg**	3	14,277	614,156	745,758	20	0	20	13	13	26	2
**Sekhukhune**	1	31,837	967,185	1,169,762	7	1	6	7	8	21	2
**Total**			**4,995,535**	**5,799,091**	**83**	**18**	**65**	**39**	**57**	**167**	**14**
**North West**											
**Dr Kenneth Kaunda**	4	45,323	628,436	742,822	76	1	75	20	21	35	7
**Bojanale**	2	43,891	1,188,457	1,657,149	50	2	48	19	21	61	5
**Ngaka Modiri Molema**	3	15,738	806,587	889,108	22	5	17	8	13	20	0
**Dr Ruth Segomotsi Mompati**	1	14,305	448,862	459,358	8	1	7	5	6	5	0
**Total**			**3,072,342**	**3,748,437**	**156**	**9**	**147**	**52**	**61**	**121**	**12**
**Gauteng**											
**Tshwane**	5	26,299	1,982,234	3,275,152	297	61	236	81	142	221	56
**Ekurhuleni**	5	27,395	2,752,678	3,379,104	251	51	200	87	138	159	28
**Sedibeng**	5	36,161	796,756	957,529	60	15	45	25	40	33	6
**City of Johannesburg**	5	25,934	3,225,309	4,949,346	478	74	404	108	182	321	54
**Westrand**	4	27,030	744,157	838,594	46	9	37	16	25	28	8
**Total**			**9,501,134**	**13,399,725**	**1132**	**210**	**922**	**317**	**527**	**762**	**152**
**Northern Cape**											
**John T Gaetsewe**	2	44,015	175,125	242,265	6	1	5	2	3	4	3
**Frances Baard**	4	12,451	325,501	387,742	23	4	19	9	13	12	6
**Pixley ka Seme**	2	29,618	164,607	195,596	8	2	6	5	7	2	0
**Namakwa**	4	16,438	108,110	115,489	7	3	4	2	5	3	1
**Z F Mgcawu**	3	28,126	210,310	252,691	2	0	2	4	4	8	3
**Total**			**983,653**	**1,193,783**	**46**	**10**	**36**	**22**	**32**	**29**	**13**
**Eastern Cape**											
**Alfred Nzo**	1	29,312	392,180	867,864	3	2	1	0	2	9	1
**O R Tambo**	1	22,007	1,676,590	1,457,384	1	1	0	0	1	19	0
**Joe Gqabi**	1	21,976	350,211	372,911	5	2	3	3	5	5	0
**Chris Hani**	1	32,933	809,582	840,054	8	5	3	6	11	7	1
**Amathole**	1	15,036	1,675,901	880,791	5	5	0	2	7	6	0
**Cacadu/Sarah Baartman**	3	43,497	388,207	479,922	10	9	1	22	31	11	1
**Nelson Mandela Bay**	5	35,796	1,028,016	1,263,051	69	66	3	41	107	54	2
**Buffalo City**	4	23,377	702,281	834,997	25	25	0	13	38	31	0
**Total**			**7,022,968**	**6,996,974**	**126**	**115**	**11**	**87**	**202**	**142**	**5**
**Western Cape**											
**Central Karoo**	4	18,264	60,483	74,247	3	0	3	1	1	2	0
**Eden**	4	25,204	454,924	611,279	34	15	19	20	35	37	7
**Overberg**	5	44,562	203,519	286,786	8	1	7	10	11	10	2
**Cape Winelands**	5	44,197	730,494	866,001	33	10	23	14	24	35	5
**City of Cape Town**	5	14,611	2,892,243	4,005,015	324	83	241	110	193	194	33
**West Coast**	5	1.00	282,673	436,403	11	4	7	10	14	16	1
**Total**			**4,624,336**	**6,279,731**	**413**	**113**	**300**	**165**	**278**	**294**	**48**

**Note**

*This South African Index of Multiple Deprivation (SAIMD) includes indicators from four domains: income and material deprivation, employment deprivation, education deprivation, and living environment deprivation, measured at either the individual or household level according to the indicator. This calculation was done in 2013/14 based on the 2011 census and was assumed to remain constant over the time period. (District Health Barometer (2016/17) [21]

*The overall SAIMD combines these individual domains of deprivation using equal weights

* The results were produced at ward level, with the most deprived ward given a rank of 1 and the least deprived a rank of 4277 [30].

*Each district was ranked according to level of deprivation and categorised into a socio-economic quintile (SEQ)

*Districts that fall into Quintile 1 (lowest quintile) are the most deprived districts. Those that fall into Quintile 5 are the least deprived (best-off) [30]

In KwaZulu-Natal most districts in Quintile 1 had marginal increases in numbers of pharmacies. The Umgungundlovu district increased by 42 pharmacies post regulation with a total of 65 located mostly within the city centre. This may be due to it being the second most populated district in the province, having both a Deprivation Index(D/I) of 2.28 and placed in Quintile 3. Despite an increase of 16 to 32 pharmacies in the Ugu district, access did not improve as new pharmacies were located where access already existed. The Ethekwini Municipality showed an improvement with most pharmacies located within or close to existing pharmacies (3.95 to 7.37 per 100,000). KwaZulu-Natal improved marginally from 3.45 to 5.43 per 100,000 population indicating the lack of growth in the rural area. The number of active pharmacies in the province increased from 315 in 2003 to 340 by 2014. In the same period 156 pharmacies closed (108 pre 2003 and 48 post 2003 registrations).

The Mpumalanga province showed the most improvement: Ehlanzeni (16 to 67), Nkangala (31 to 63) and Gert Sibanda (32 to 51). All three districts have large populations and are classified in Quintiles 3 and 4. There has been growth both in the city and regional service centres as well as in the populated rural areas especially in Ehlanzeni. The province started from a low base of 2.53 per 100,000 population and improved to 4.71 per 100,000.

Limpopo province grew by 13% and showed the best new pharmacy growth (10.67%) gaining approximately 13 pharmacies per year. All districts showed improvement in the number of pharmacies in both city and densely populated rural areas. Most districts have a large population base of over a million persons. The Capricorn district improved from 1.56 to 3.98 pharmacies per 100,000. Overall, the province saw an improvement from 1.14 to 3.55 pharmacies per 100,000 population. While new pharmacies showed a comparatively low closure rate (7.7%), the combined closure of pre and post 2003 pharmacies was 13.45% between 2004 and 2014.

The North West district of Bojanale with a population of 1.66 million people showed a marked increase in the number of pharmacies post 2003 growing from 21 to 80 active pharmacies in 2014 with a growth from 1.76 to 4.83 per 100,000 population. The Dr. Kenneth Kaunda district also showed improvement from 21 to 55 pharmacies primarily in the urban centres. The North West province gained a net of 10 pharmacies per year since the regulations growing from 1.99 to 4.62 per 100,000 population.

Three districts in the Gauteng province (Tshwane 221, Ekurhuleni 159, and City of Johannesburg 321) showed a large increase in number of new pharmacies. The data indicates an increase in the number of pharmacies per 100,000 population (Tshwane 4.08 to 9.22, Ekurhuleni 3.16 to 7.28, City of Johannesburg 3.34 to 8.67). The smaller districts such as Sedibeng (3.1 to 6.06) and Westrand (2.1 to 5.24) also showed increases. The province moved from 5.52 to 7.99 per 100,000 population. The rate of closure of pharmacies was 25.1% between 2004 to 2014 with new pharmacies experiencing a lower closure rate of 16.6% compared to a 39.8% closure rate from the pre 2003 pharmacies showing a reduction from a 527 in 2004 to 317 in 2014.

The Northern Cape showed a marginal increase from 3.25 to 4.27 per 100,000 with none of the districts showing significant increases. Frances Baard showed a slight improvement from 2.76 to 5.42 per 100,000 population although 33.3% of new pharmacies that opened after regulations closed by 2014. There was low growth of 1.73 pharmacies per year contributing marginally (1.36%) to the overall growth of pharmacies in South Africa with 31% of all new pharmacies closing during the period 2004 to 2014.

Pharmacies that were registered pre − 2003 in the Eastern Cape districts of Alfred Nzo and OR Tambo all closed by 2014 with only 9 and 19 respectively still active post 2003 registrations. The economic hubs of Nelson Mandela Bay and Buffalo City showed improvement in the cities and large regional centres increasing from 3.9 to 7.52 and 1.85 to 5.27 respectively per 100,000 population. The closure of new pharmacies in the Eastern Cape was low at 3.4% (5 of 147). By 2014 56.0% of active pharmacies in 2004 had closed leaving the province with 229 pharmacies in 2014 (87 + 142).

Western Cape increased from 5.77 to 9.55 per 100,000 population with the City of Cape Town showing a marked improvement of 3.8 to 7.59 per 100,000 population mostly in the city and large regional centres. Also evident was the dominance of corporate pharmacy (150 new openings) compared to 144 independents. The Central Karoo and Overberg area showed little improvement with other districts improving only marginally. The province showed an average attrition rate of new pharmacies of 14%. Approximately 66.37% of new pharmacies opened in the City of Cape Town with the bulk of the balance being shared between Eden (12.7%) and the Cape Winelands (11.7%).

A summary of all the active pharmacies per province in 2014 (3021) is presented in Table 3 below. Of these, 2019 pharmacies (66.8%) opened after the regulation with Gauteng, Western Cape and KwaZulu-Natal showing increased new openings.

**Table 3:** Active Registered Pharmacies in 2014

Active Pharmacies in 2014						
	Independents	Corporate	Total	Pre 2003 registrations	Total Active	% Growth (New)
**Eastern Cape**	94	48	142	87	229	7.03
**Free State**	62	31	93	51	144	4.61
**Kwa Zulu-Natal**	192	100	292	207	499	14.46
**Mpumalanga**	91	28	119	62	181	5.89
**Limpopo**	148	19	167	39	206	8.27
**North West**	84	37	121	52	173	5.99
**Gauteng**	472	290	762	317	1079	37.74
**Northern Cape**	16	13	29	22	51	1.44
**Western Cape**	144	150	294	165	459	14.56
**TOTAL**	**1303**	**716**	**2019**	**1002**	**3021**	**100.00**

## Discussion

The increase in the number of pharmacies post regulations from 1624 in 2004 to 3021 in 2014 (Table 3) is in keeping with the OBIG 2006 European [10] study which showed that there was an increase in the number of pharmacies in countries that had introduced liberalisation. Norway has 8500 [22] inhabitants per pharmacy with the regulated Spain (2050) and Austria (3700). Ireland, a deregulated zone has 3000 inhabitants per pharmacy. South Africa moved from 28,000 to 18,000 inhabitants per pharmacy, short of the acceptable international standards.

Most growth of new pharmacies occurred in Gauteng, KwaZulu-Natal and Western Cape. These provinces contain the major metropolitan areas; Tshwane, Ekurhuleni, City of Johannesburg; Ethekwini; and City of Cape Town. These five large districts obtained 52% of all new pharmacies. This urban clustering and lack of improvement in rural areas is in keeping with local [6, 14] and international [10, 11] study findings. Areas with the highest deprivation had fewer pharmacies per 100,000 population. Within the framework of current legislation South Africa must find a way to incentivise the opening of pharmacies in areas of need.

After Norway’s [10] deregulation in 2001 every second municipality had no pharmacy. Urban clustering, vertical integration and chain ownership by wholesalers resulted in 4 of 5 pharmacies being owned by 1 of 3 chains. Pharmacists own only 19% of Norwegian pharmacies. The Norwegian experience led researchers to believe that deregulation leads to market dominance and minimises competition. Principle areas of practice in Europe are 78.5% in community, 8.9% in hospital and 12.6% in other areas [23]. In South Africa 68.3% of registered pharmacists practiced in the community sector in 2014 [24]. Any regulation must be carefully monitored to ensure stability and job security in this market. Deregulation in most countries [14] results in corporatisation of community pharmacy. In South Africa following deregulation 35% of new pharmacies were corporate listed. Similarly, Norway (96%), Sweden (86%), US (64%), and United Kingdom (UK) (61%) showed dominance of corporatisation post deregulation [14, 25].

In Sweden the Agency for Growth Policy Analysis (Ministry of Enterprise, Energy and Communication) found that after deregulation, new pharmacies opened in urban and not rural areas, and the price of over-the-counter medicines did not decrease [26]. Lluch and Kanavos [27] highlighted the risk associated with chains and vertical integration leading to monopoly. Policies addressing these risks should be considered.

The study does have limitations. The pharmaceutical service per population ratio is only reflective for community pharmacy and excludes the public sector. The type of ownership was restricted to independent and corporate pharmacy only. The primary source document which was the Council register had inaccuracies as well as insufficient ownership data. The study did not look into quality of service provided, or operational efficiencies.

Future research should include investigating:

- means of improving “rural policy, rural health services and rural practice [28]”
- The cost implication of the disruption of existing pharmacies in terms of capital and infrastructure loss
- the implications of concentration of pharmacy staff within the same location for service delivery in areas of need
- the long term impact on pharmacy skills development as new pharmacists are forced into prematurely taking on responsible pharmacist roles [13, 29]
- the overall cost of pharmaceutical care in respect of duplication as opposed to rationalization of resources
- benchmark indicators of accessibility, quality and expenditure, which ranks better in strict regulated environments than in the non-regulated countries [16]

## Conclusions

While liberalisation laws in South Africa may have increased the number of pharmacies, it did not result in a large increase in pharmacy access in previously disadvantaged and rural areas. There is a gradual shift from independent pharmacist to corporate ownership. Other incentives and policies are required to improve access to disadvantaged areas.

## Supplementary information

**Additional file 1.** Active pharmacies in the eastern cape: registered pre-regulation.

## Data Availability

The data supporting the conclusions in this article are included within the article in the tables and figures.

## Abbreviations

GPS: Global Positioning System
FIP: International Pharmaceutical Federation
OBIG: Österreichisches Bundesinstitut für Gesundheitswesen
StatsSA: Statistics South Africa
UK: United Kingdom
US: United States
