# Supplementary material for: To evaluate the impact of opening up ownership of pharmacies in South Africa
Source: J Pharm Policy Pract. 2020 Aug 7;13:28. doi: 10.1186/s40545-020-00232-4 (PMC7412837; doi:10.1186/s40545-020-00232-4)

ACTIVE PHARMACIES IN THE EASTERN CAPE: REGISTERED PRE-REGULATION

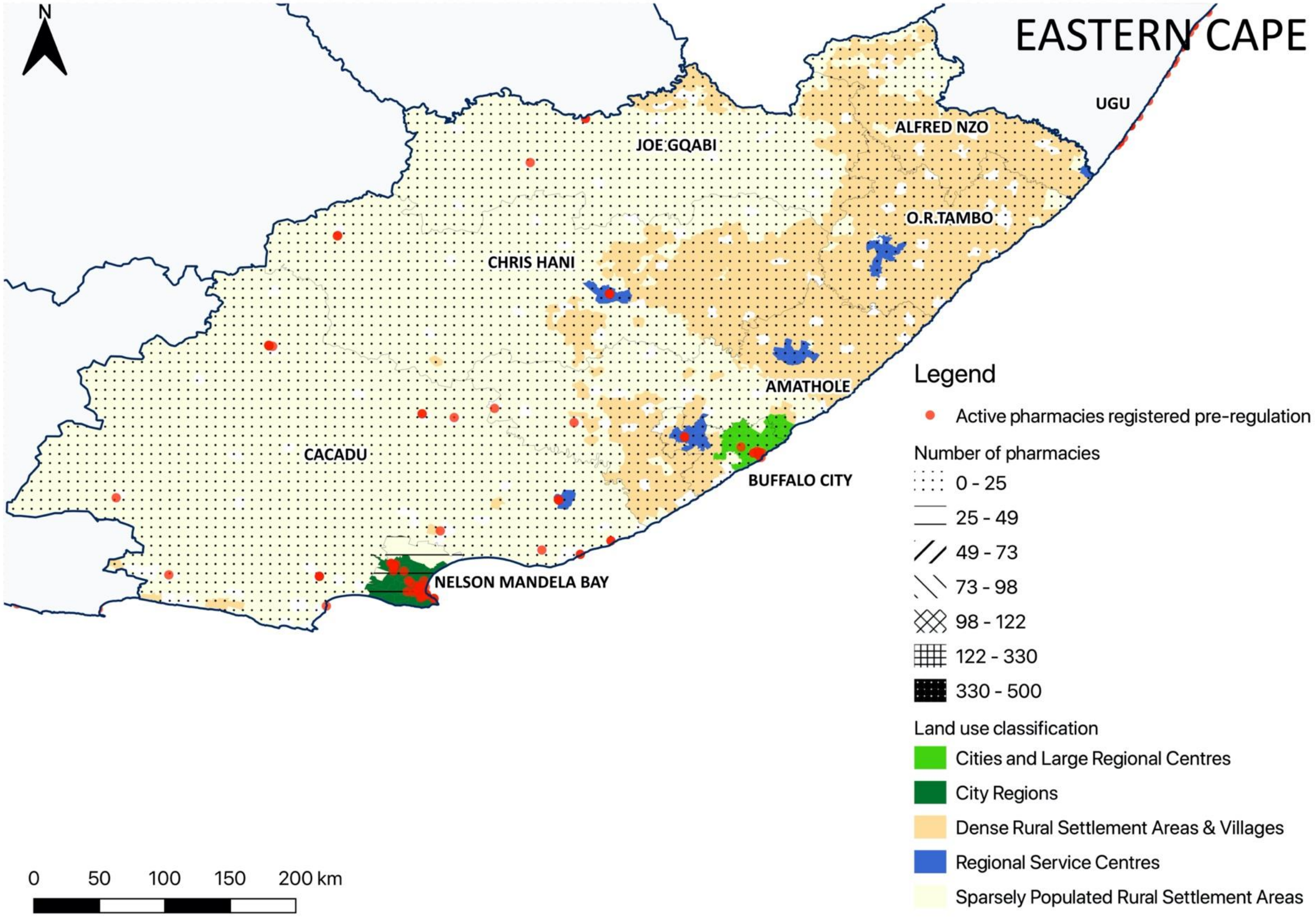

ACTIVE PHARMACIES IN THE EASTERN CAPE: PRE-REGULATION VERSUS POST-REGULATION

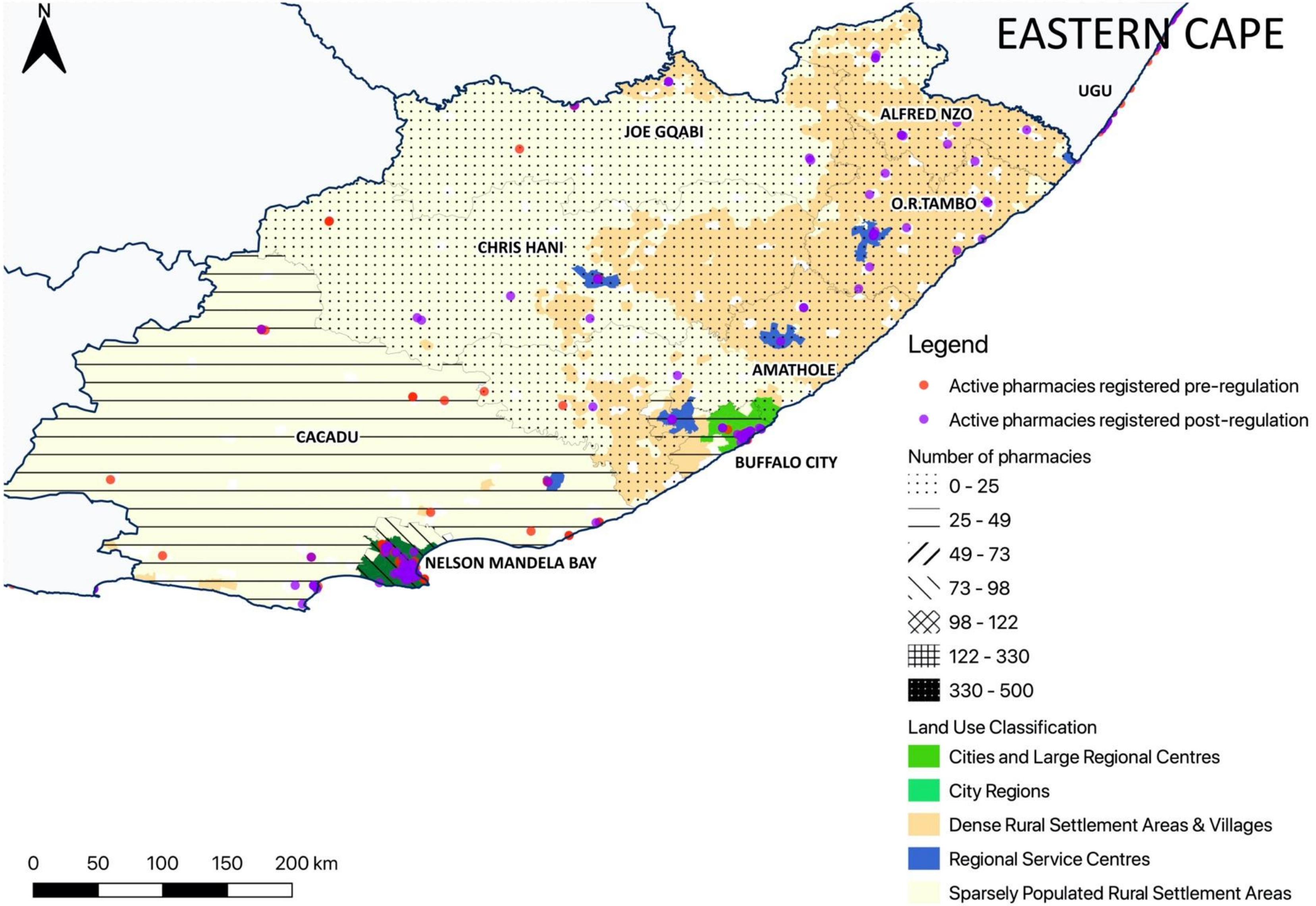

INACTIVE PHARMACIES IN THE EASTERN CAPE: POST-REGULATION CLOSURES

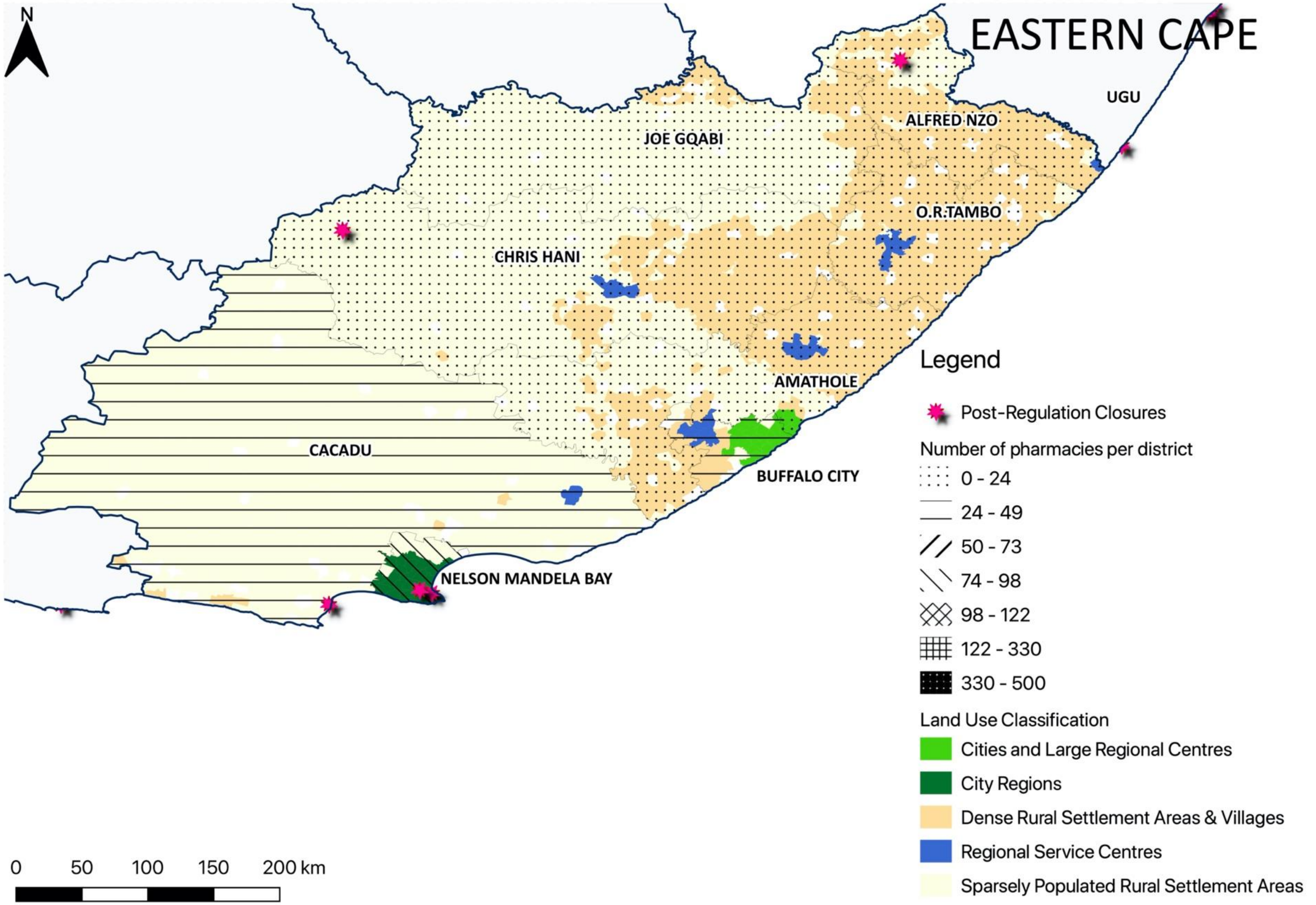

ACTIVE PHARMACIES IN THE FREE STATE: REGISTERED PRE-REGULATION

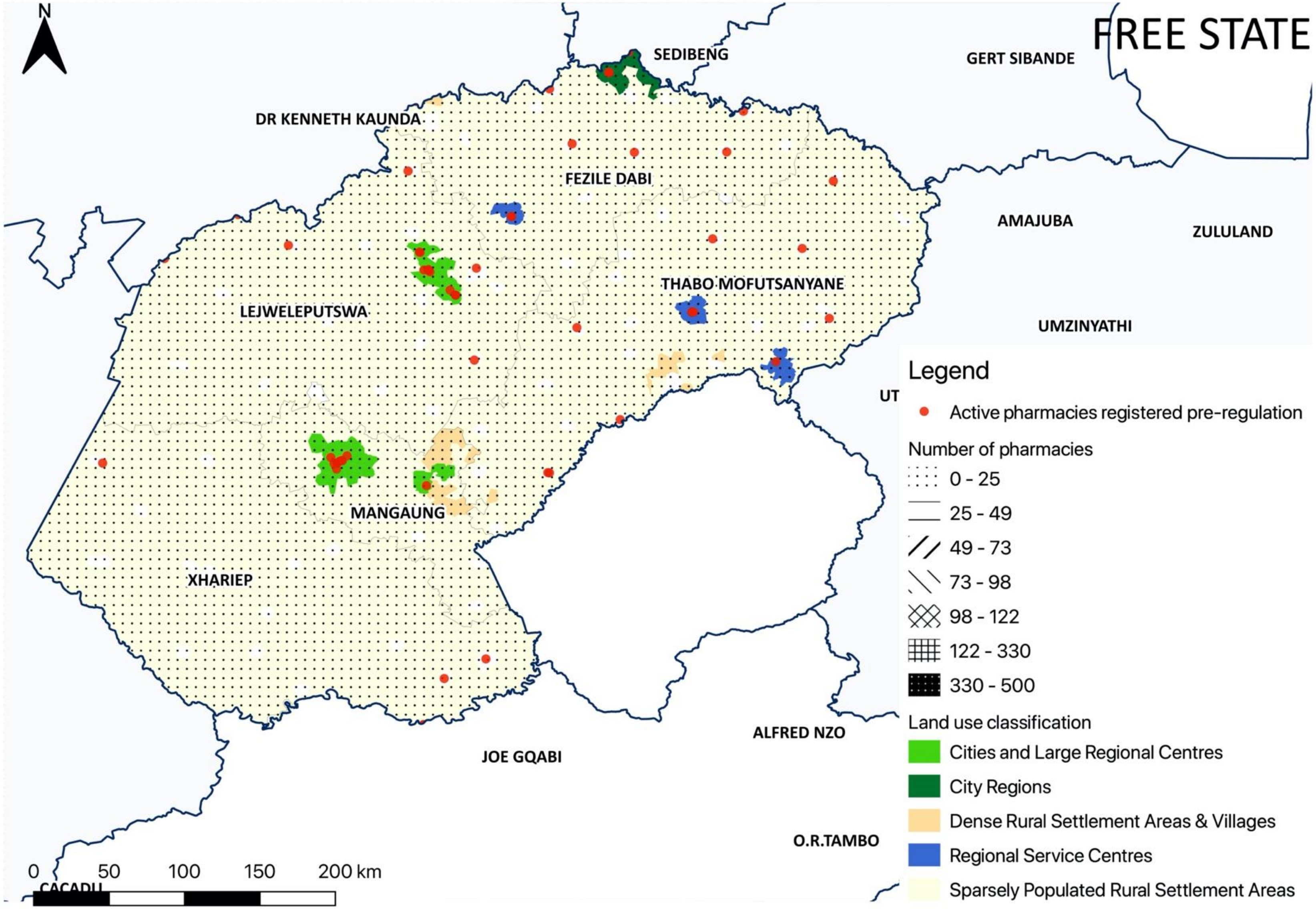

ACTIVE PHARMACIES IN THE FREE STATE: PRE-REGULATION VERSUS POST-REGULATION

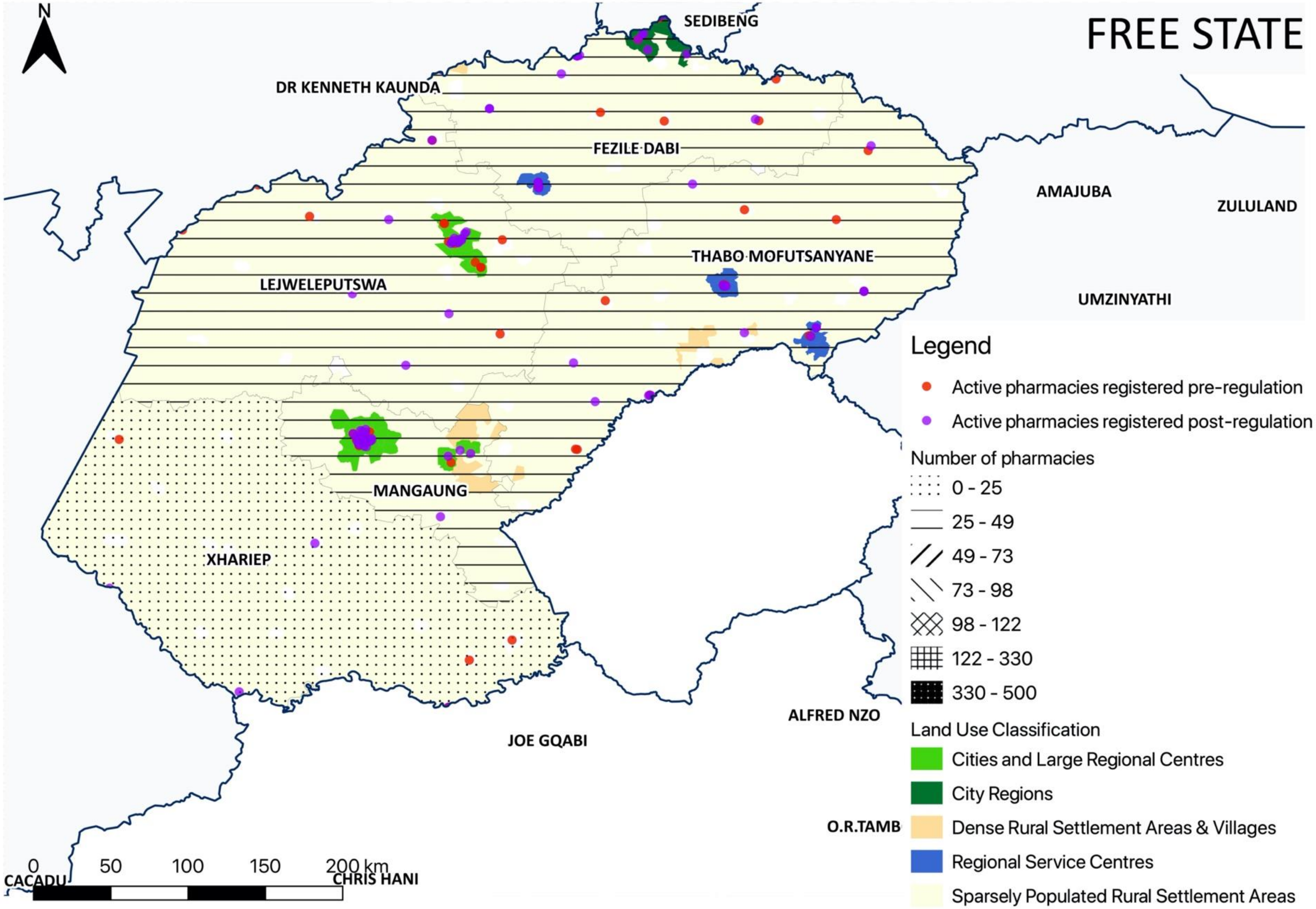

INACTIVE PHARMACIES IN THE FREE STATE: POST-REGULATION CLOSURES

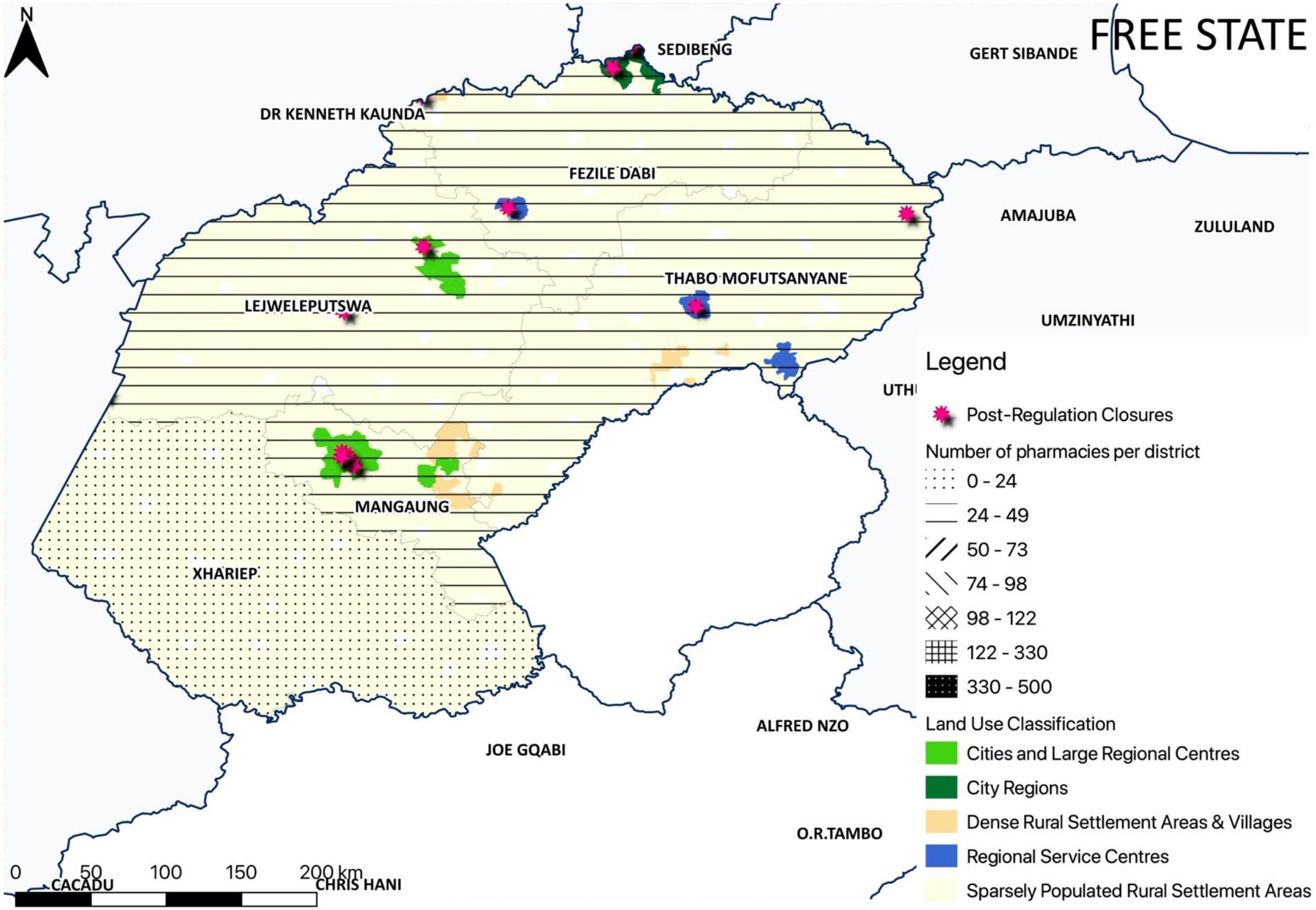

ACTIVE PHARMACIES IN GAUTENG: REGISTERED PRE-REGULATION

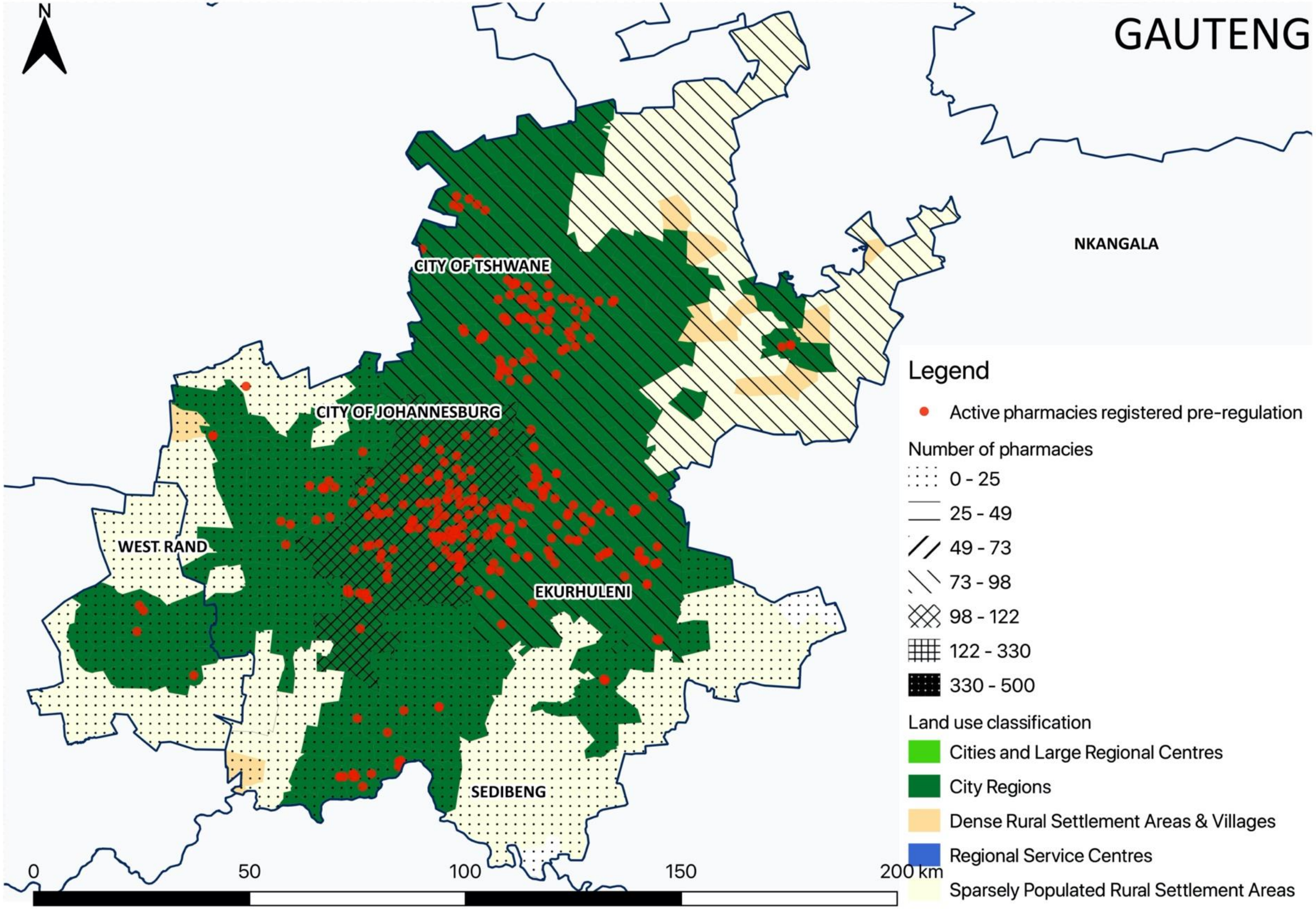

ACTIVE PHARMACIES IN GAUTENG: PRE-REGULATION VERSUS POST-REGULATION

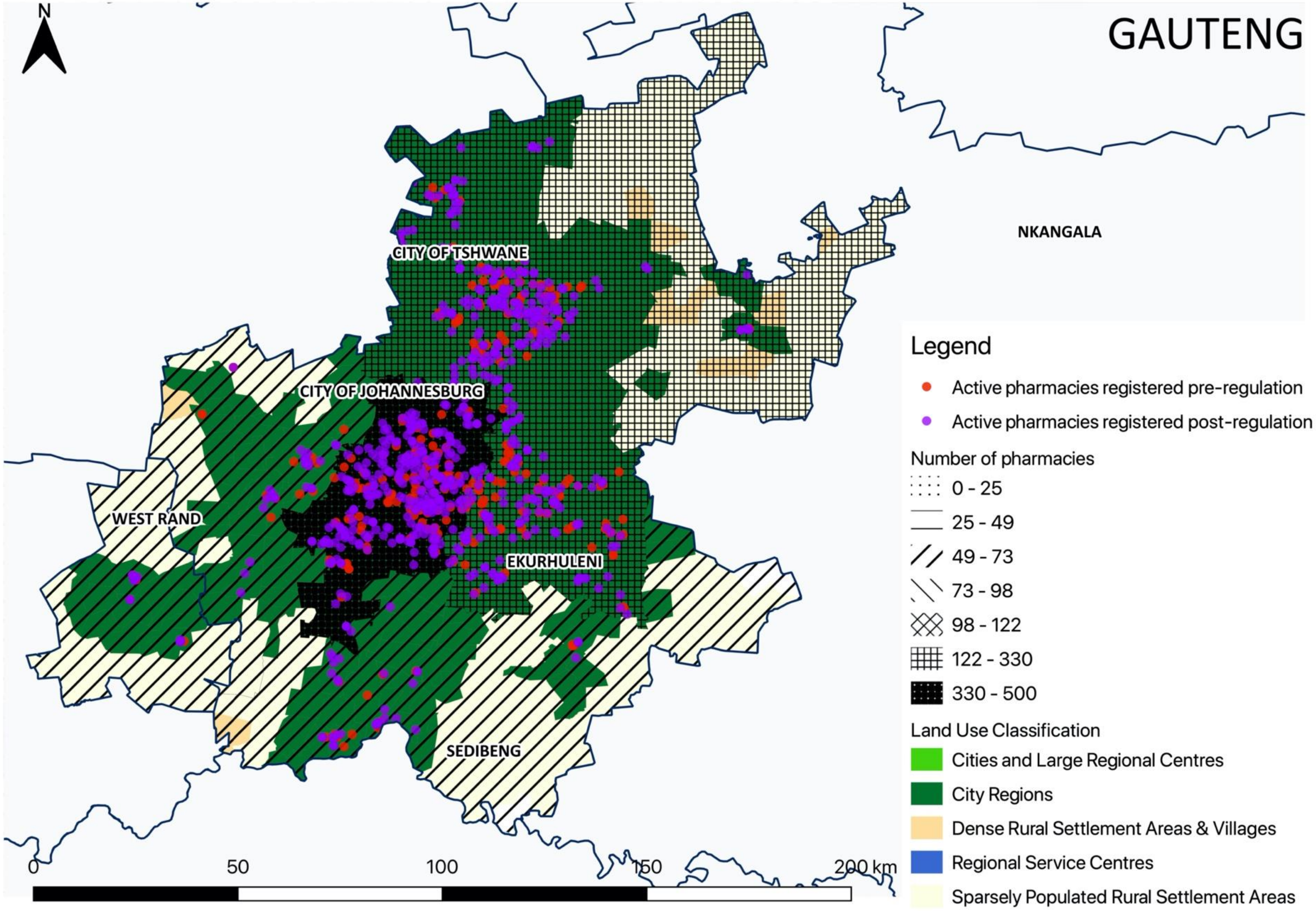

INACTIVE PHARMACIES IN GAUTENG: POST-REGULATION CLOSURES

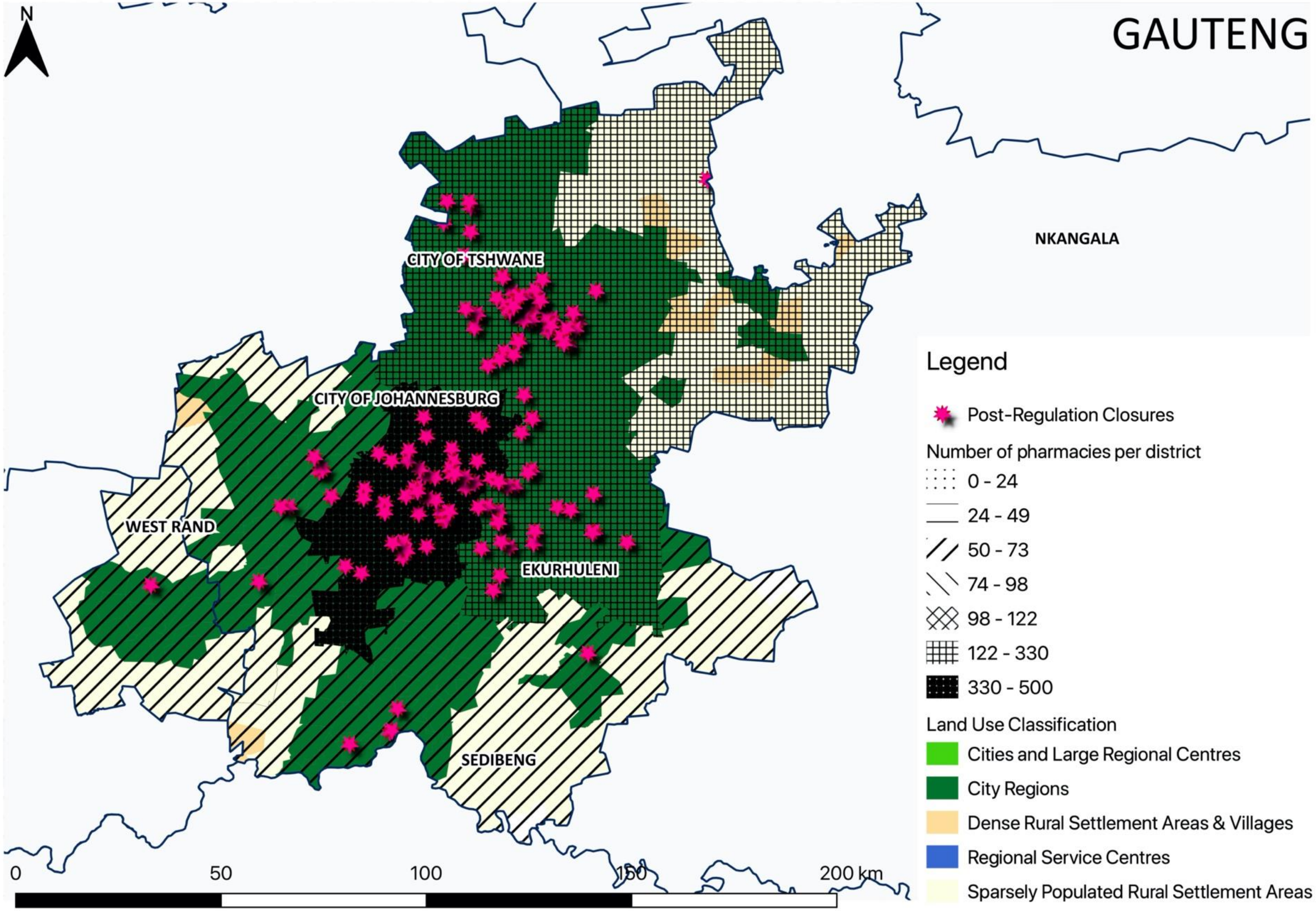

ACTIVE PHARMACIES IN KWAZULU-NATAL: REGISTERED PRE-REGULATION

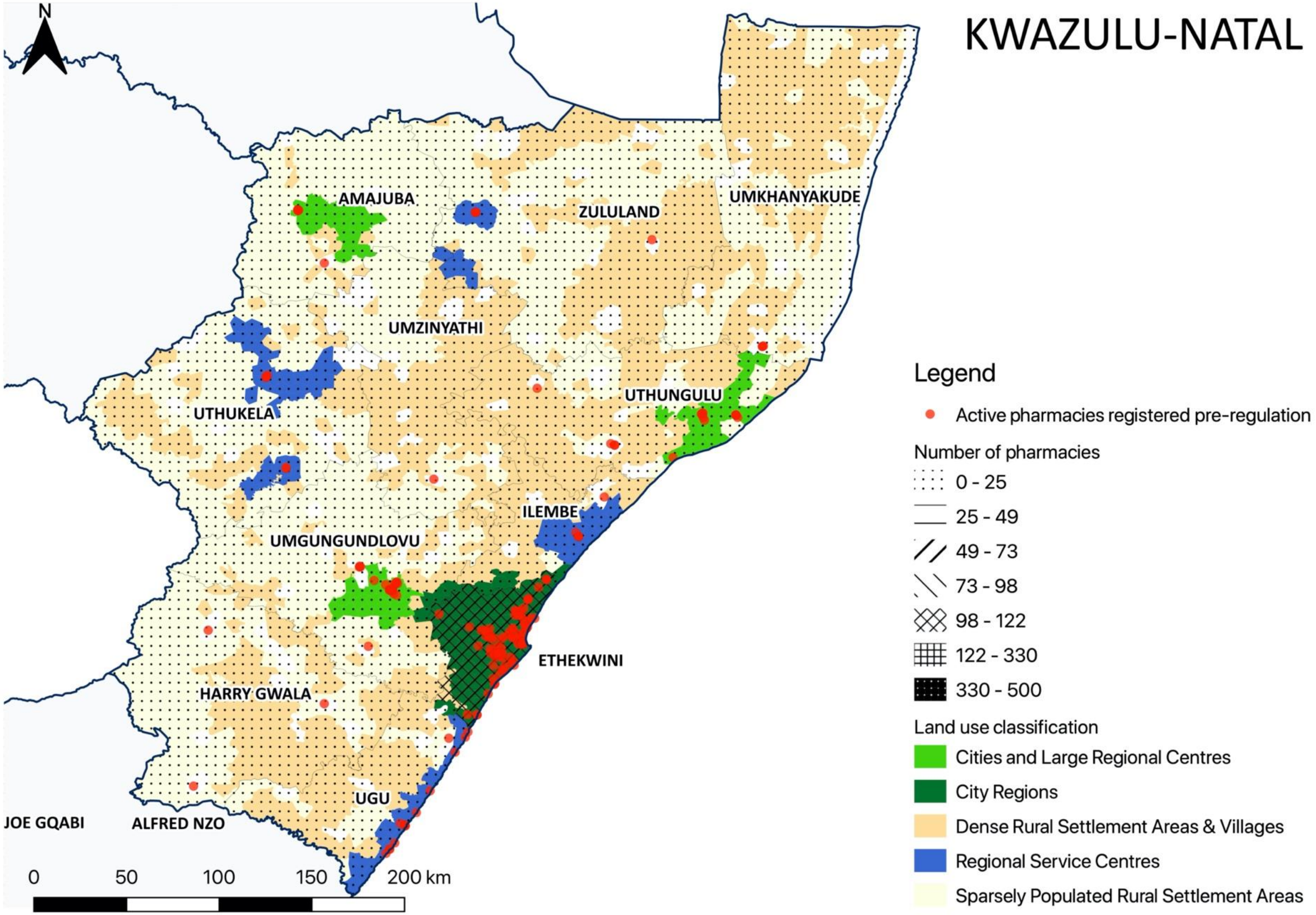

ACTIVE PHARMACIES IN KWAZULU-NATAL: PRE-REGULATION VERSUS POST-REGULATION

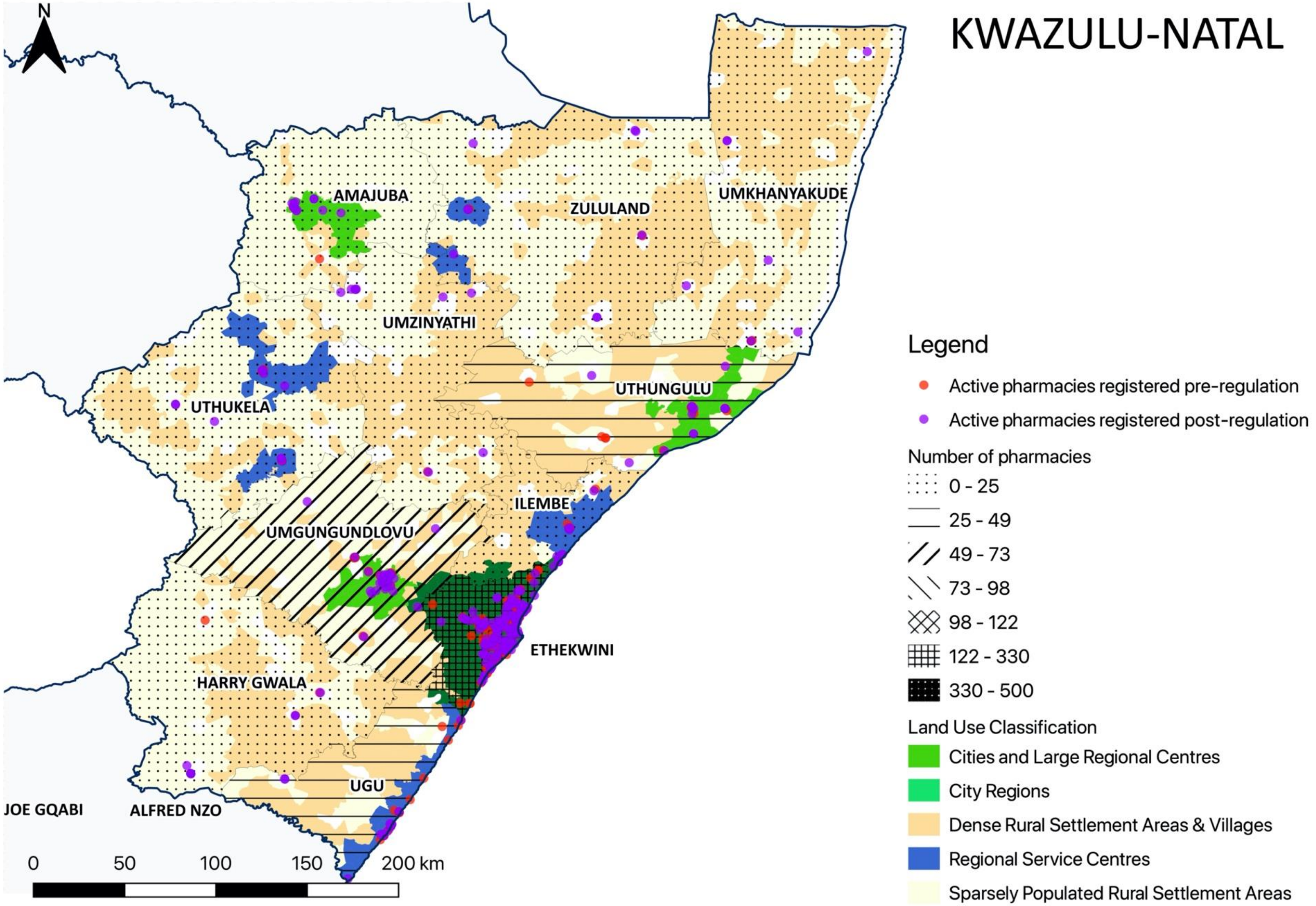

INACTIVE PHARMACIES IN KWAZULU-NATAL: POST-REGULATION CLOSURES

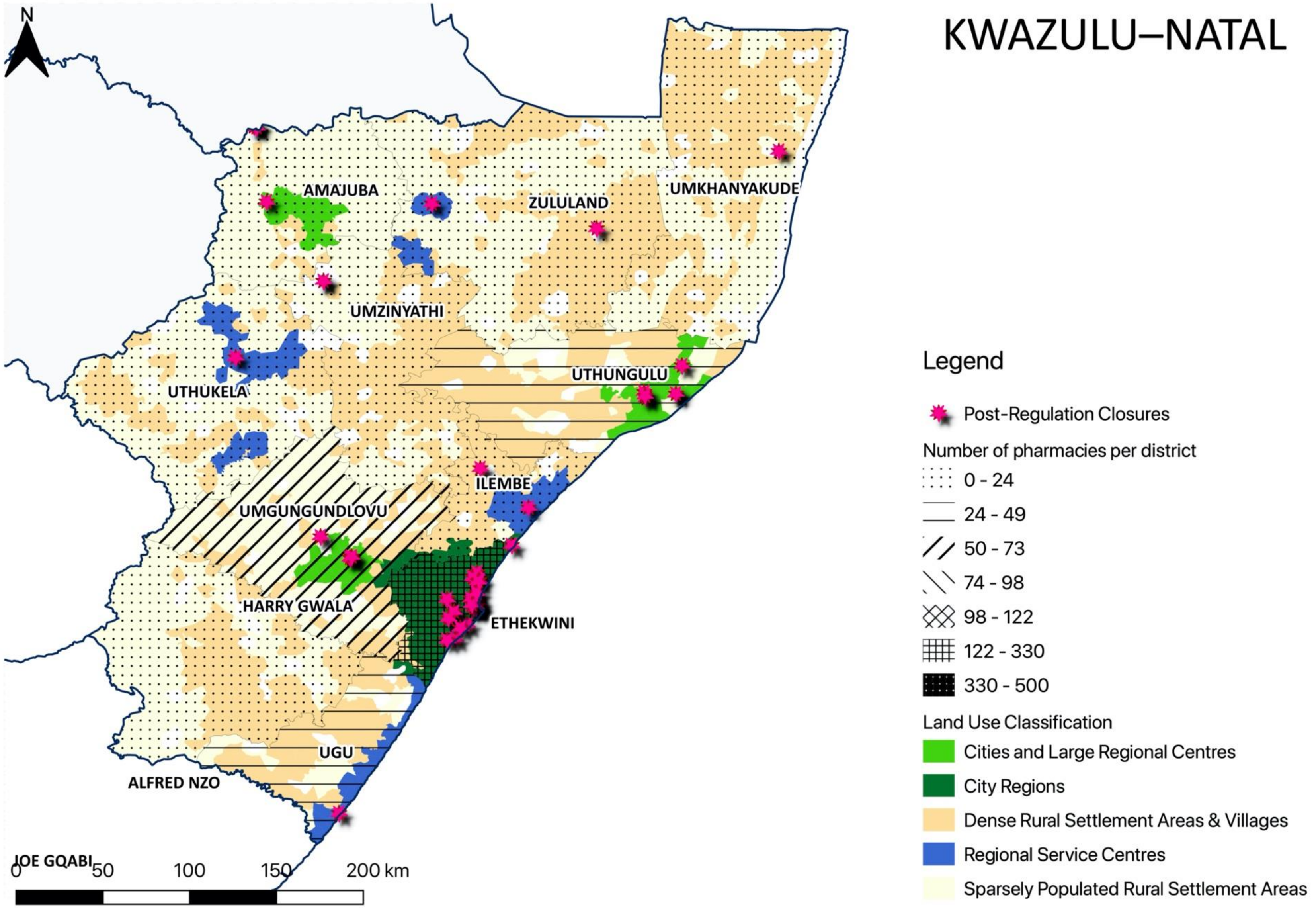

ACTIVE PHARMACIES IN LIMPOPO: REGISTERED PRE-REGULATION

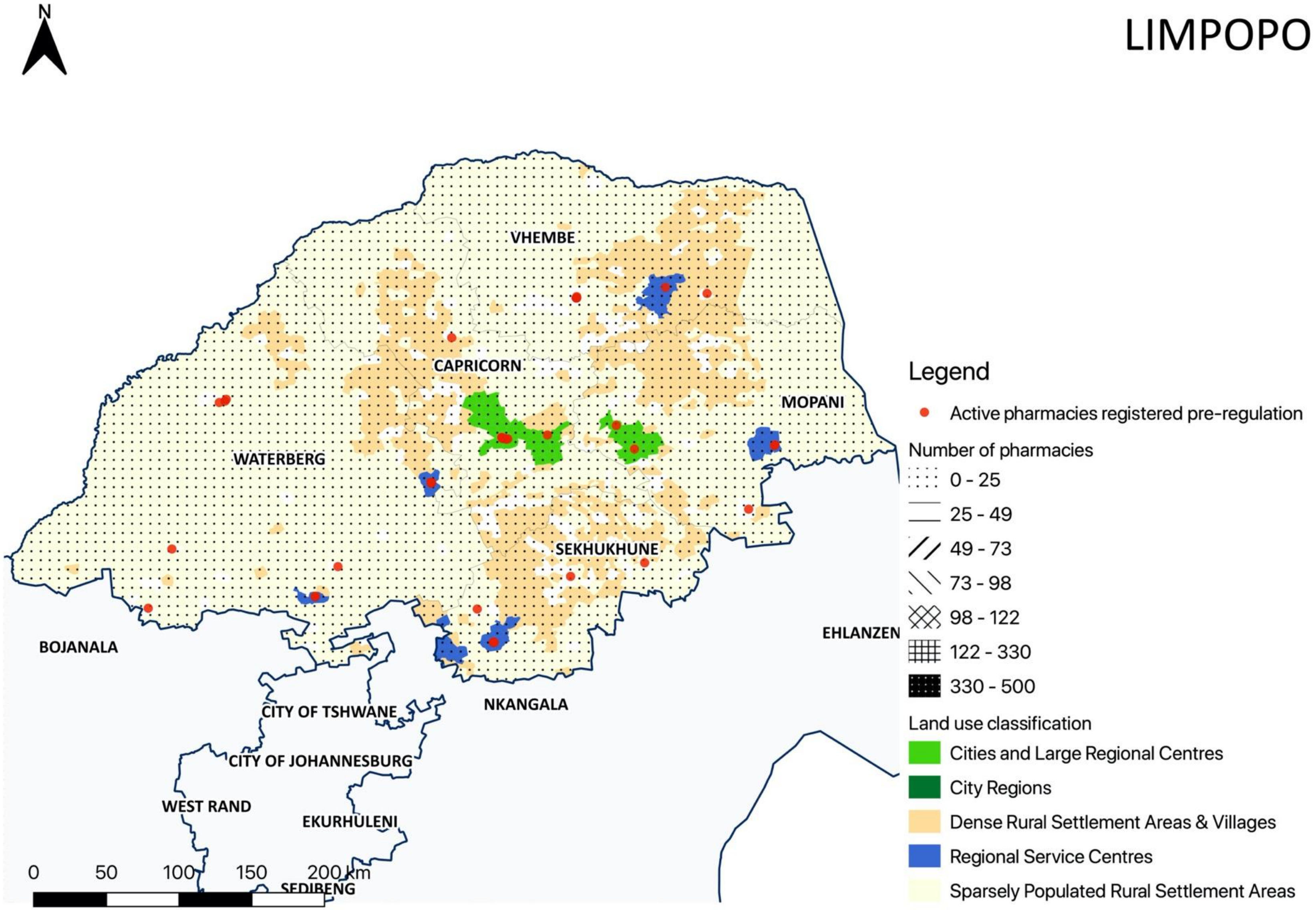

ACTIVE PHARMACIES IN LIMPOPO: PRE-REGULATION VERSUS POST-REGULATION

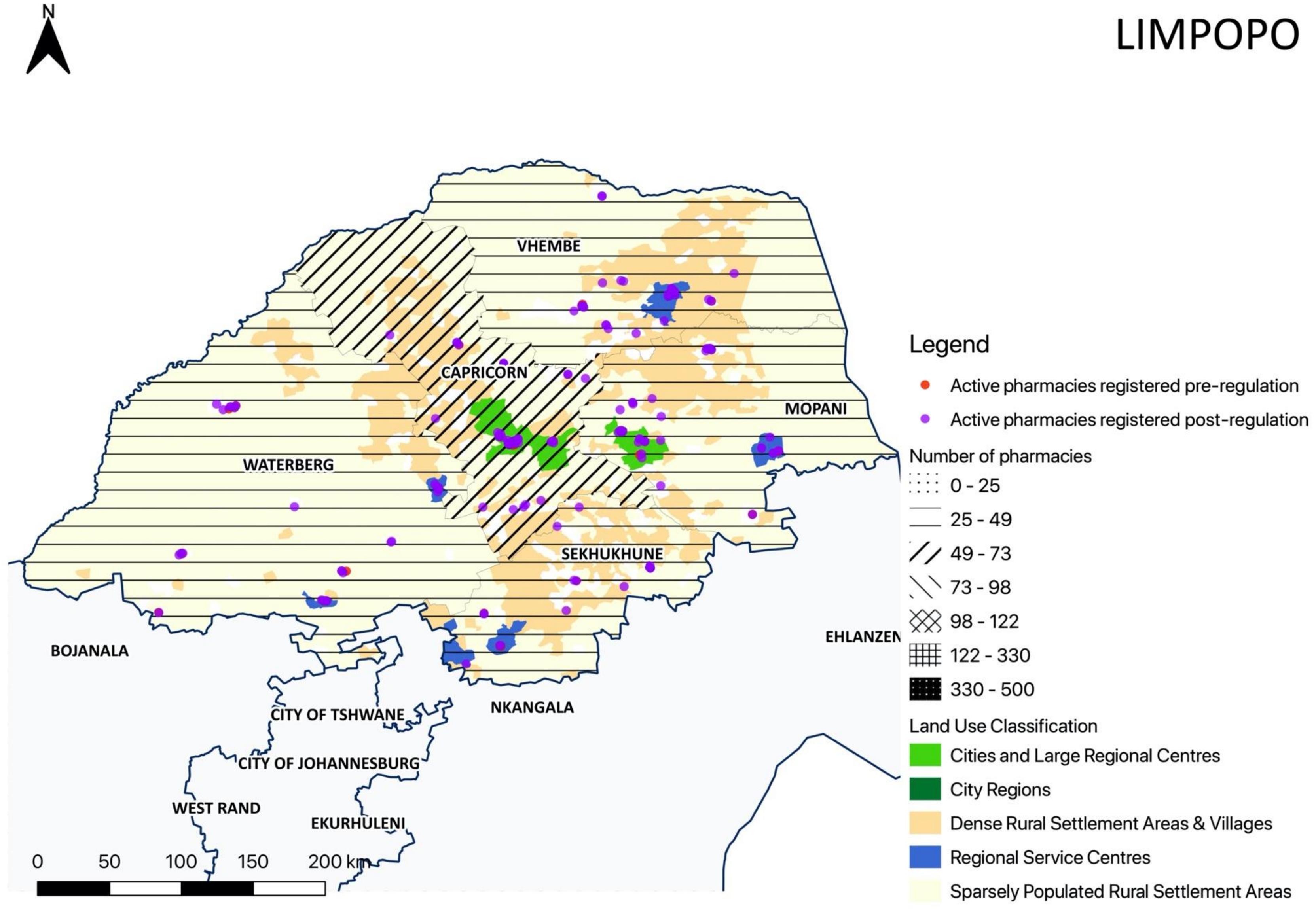

INACTIVE PHARMACIES IN LIMPOPO: POST-REGULATION CLOSURES

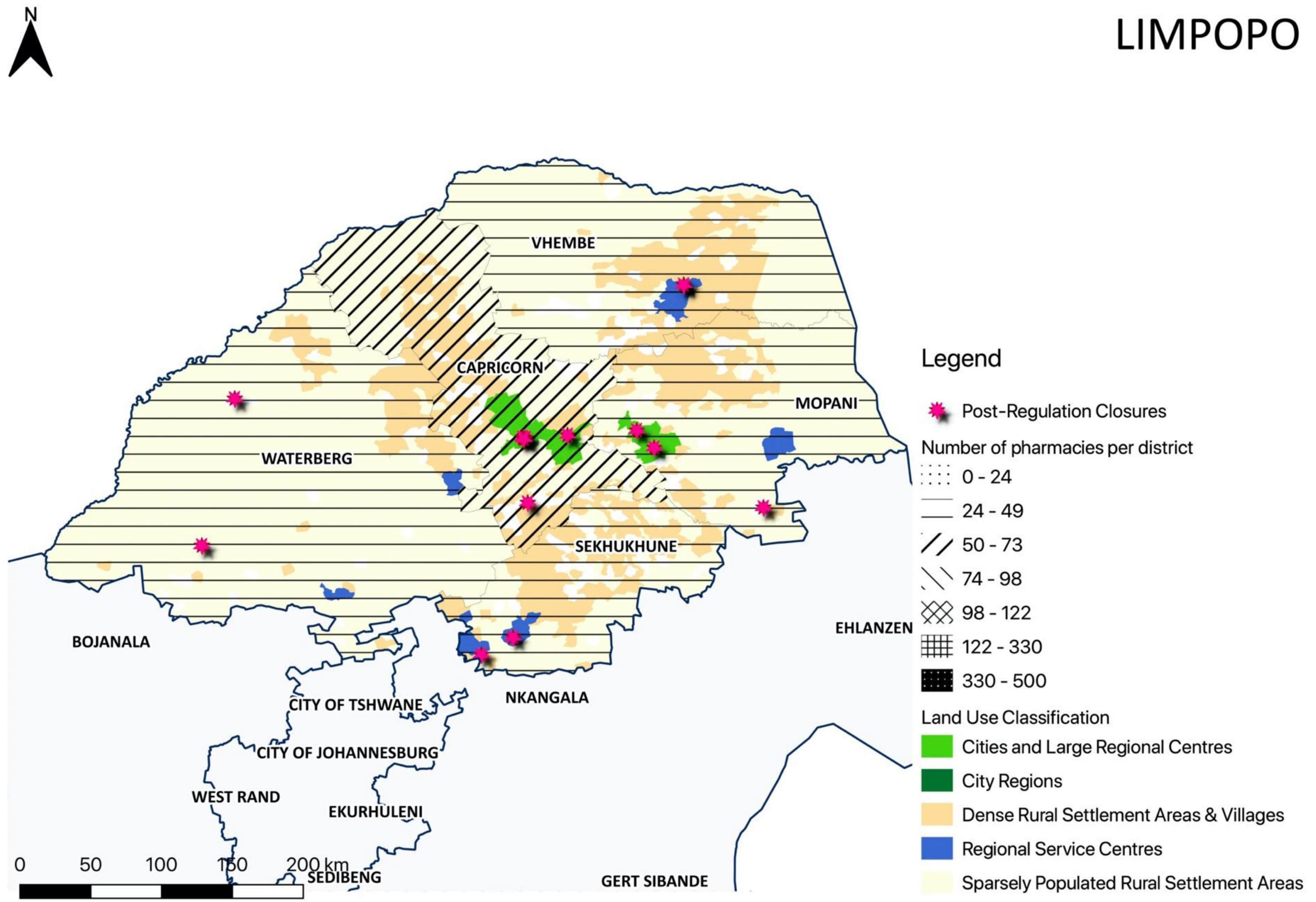

ACTIVE PHARMACIES IN MPUMALANGA: REGISTERED PRE-REGULATION

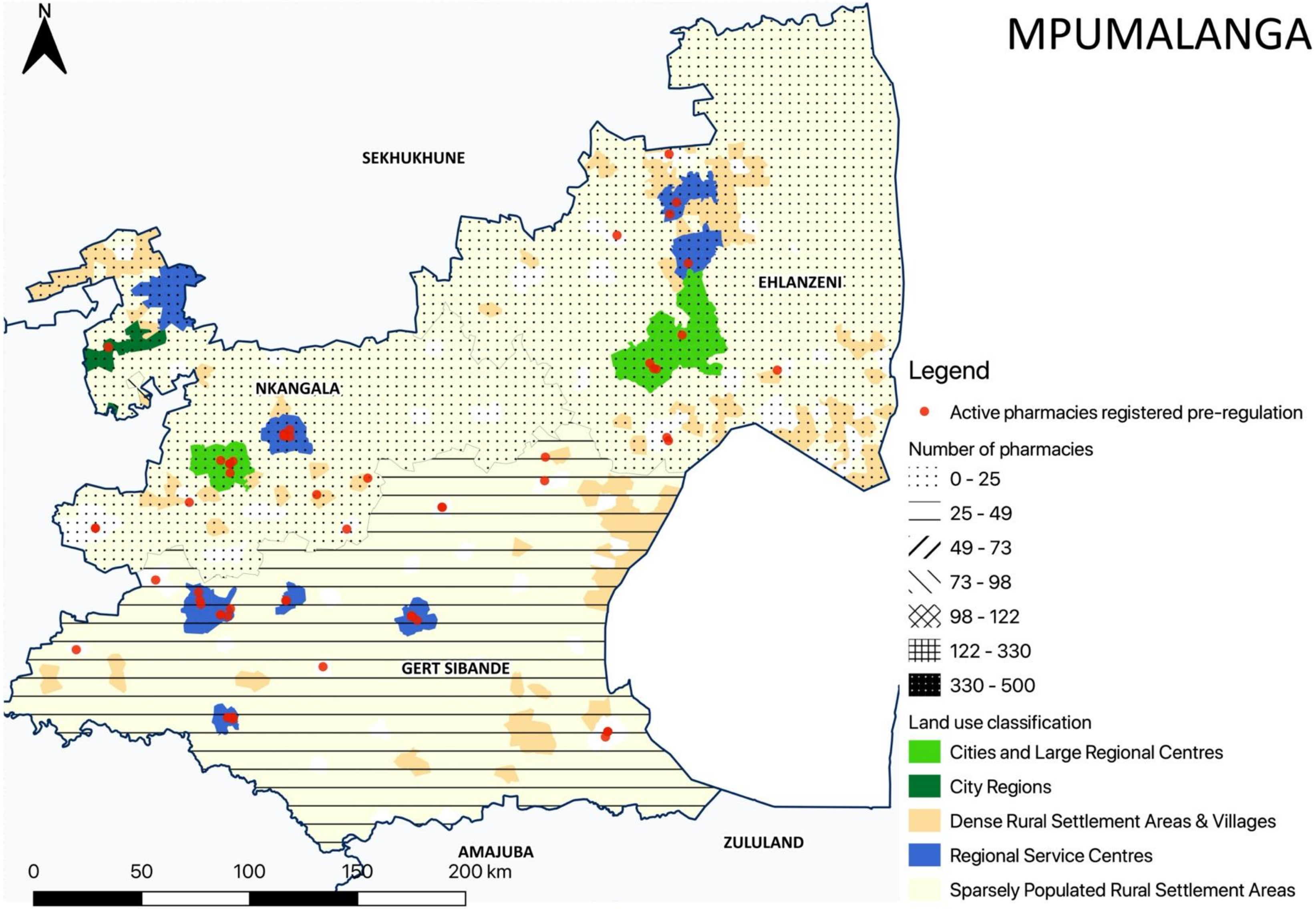

ACTIVE PHARMACIES IN MPUMALANGA: PRE-REGULATION VERSUS POST-REGULATION

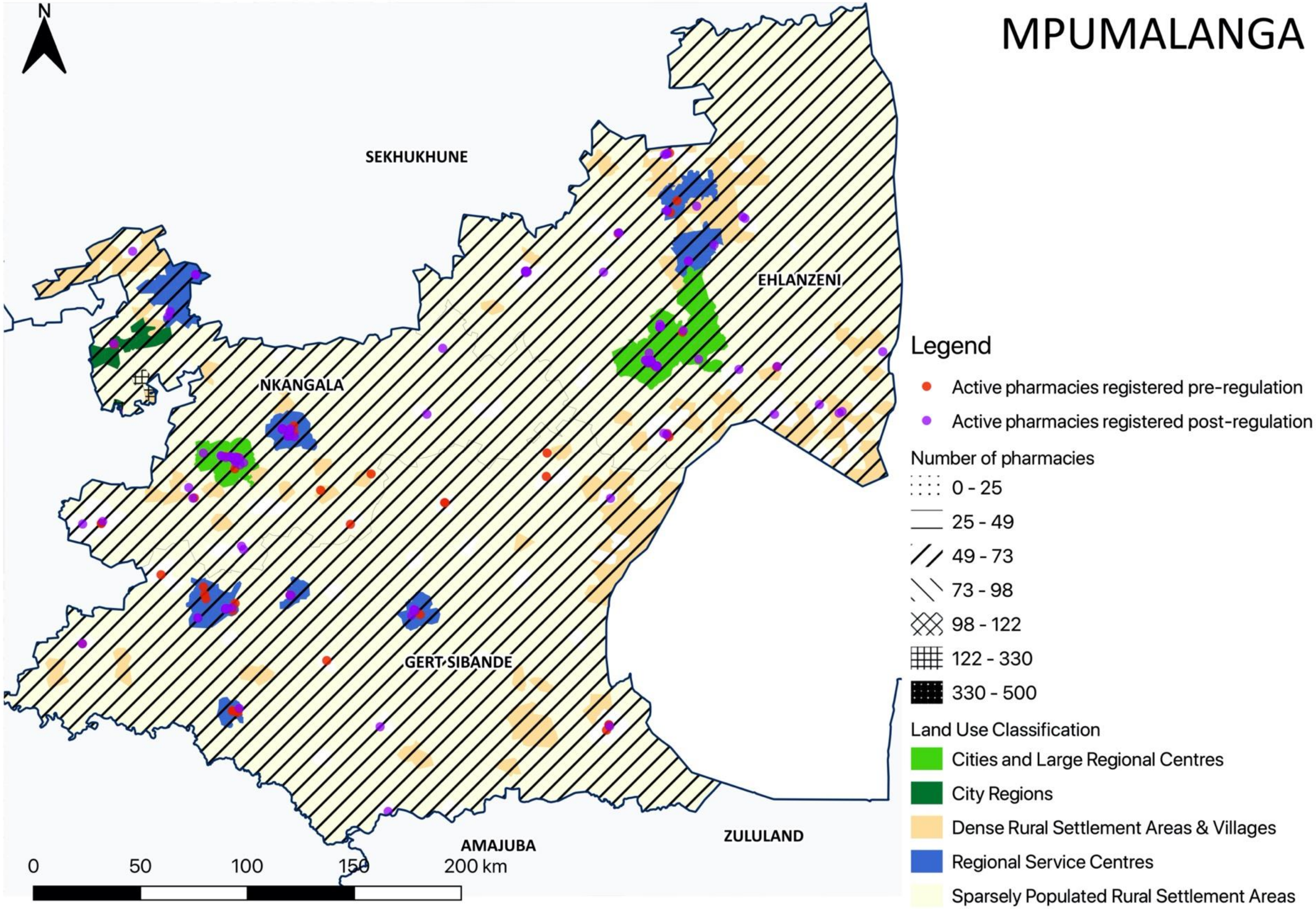

INACTIVE PHARMACIES IN MPUMALANGA: POST-REGULATION CLOSURES

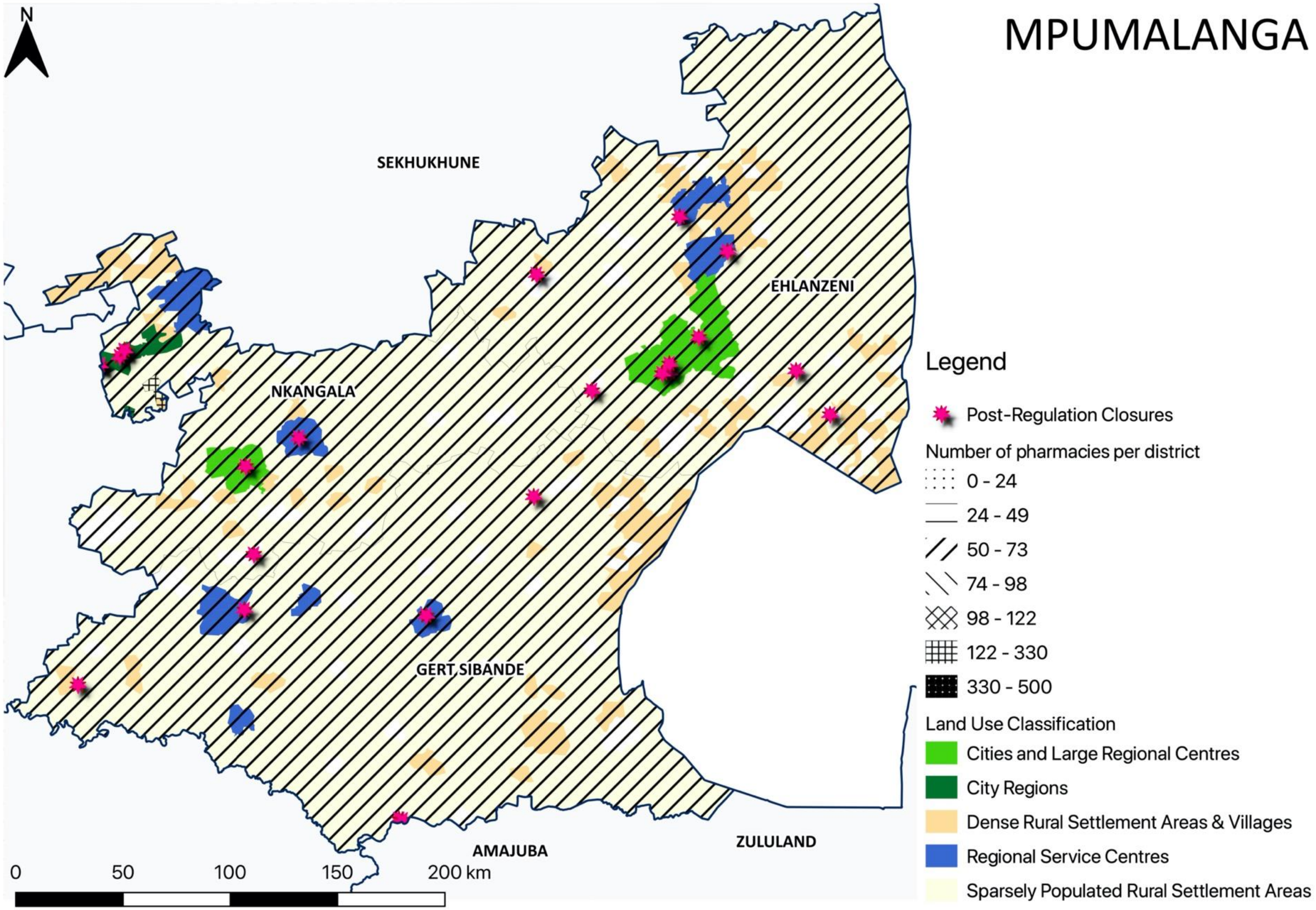

ACTIVE PHARMACIES IN THE NORTHERN CAPE: REGISTERED PRE-REGULATION

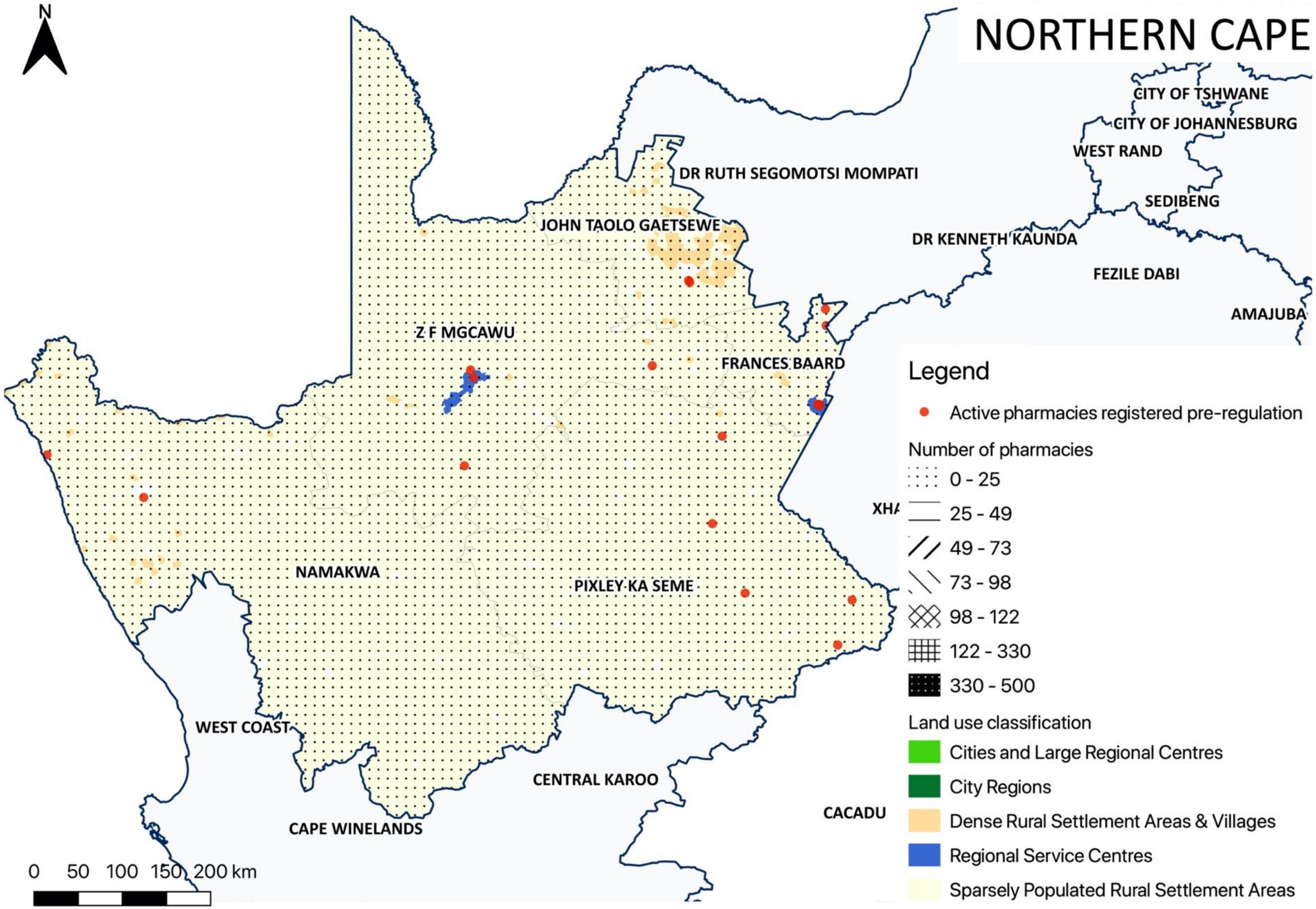

ACTIVE PHARMACIES IN THE NORTHERN CAPE: PRE-REGULATION VERSUS POST-REGULATION

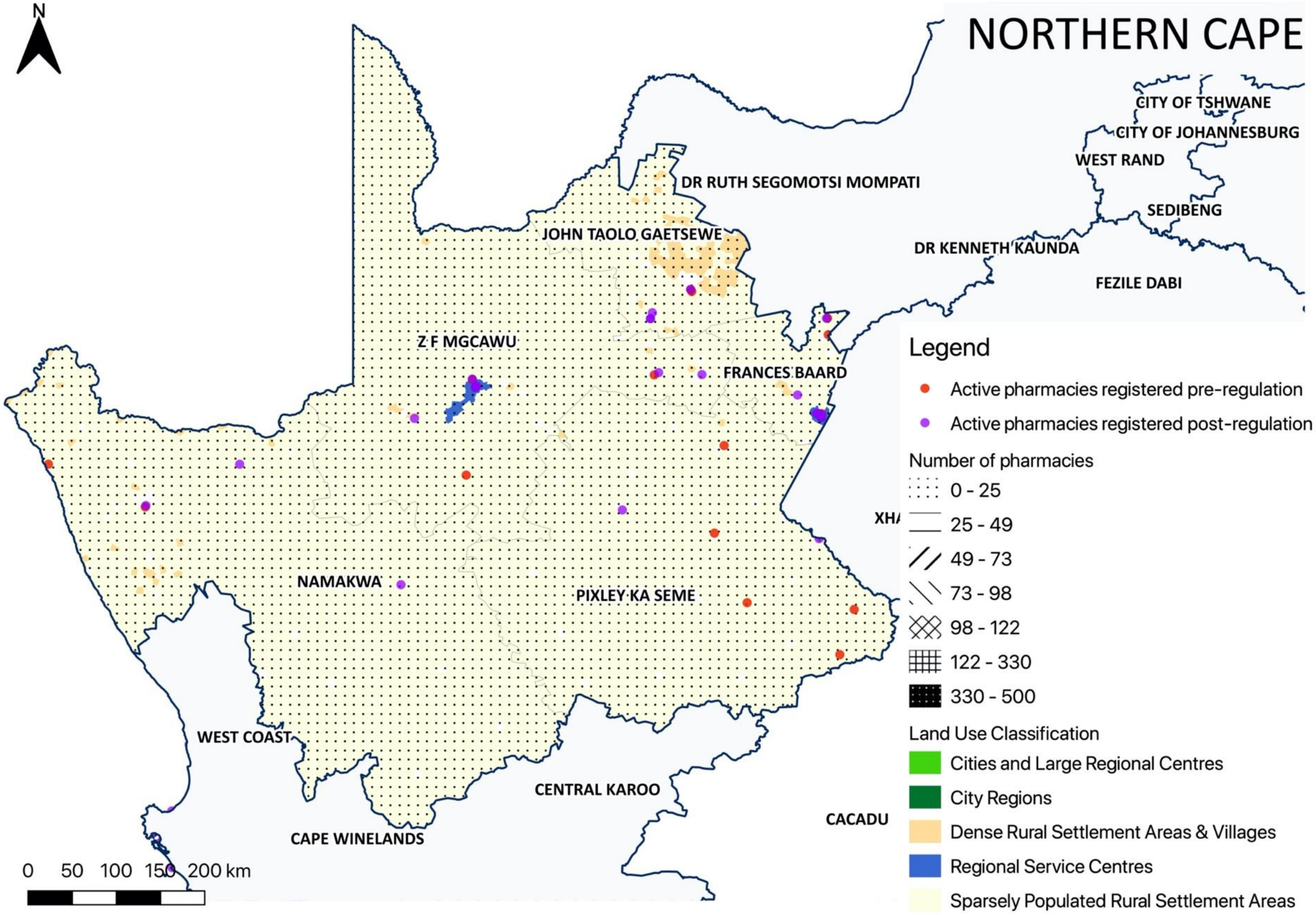

INACTIVE PHARMACIES IN THE NORTHERN CAPE: POST-REGULATION CLOSURES

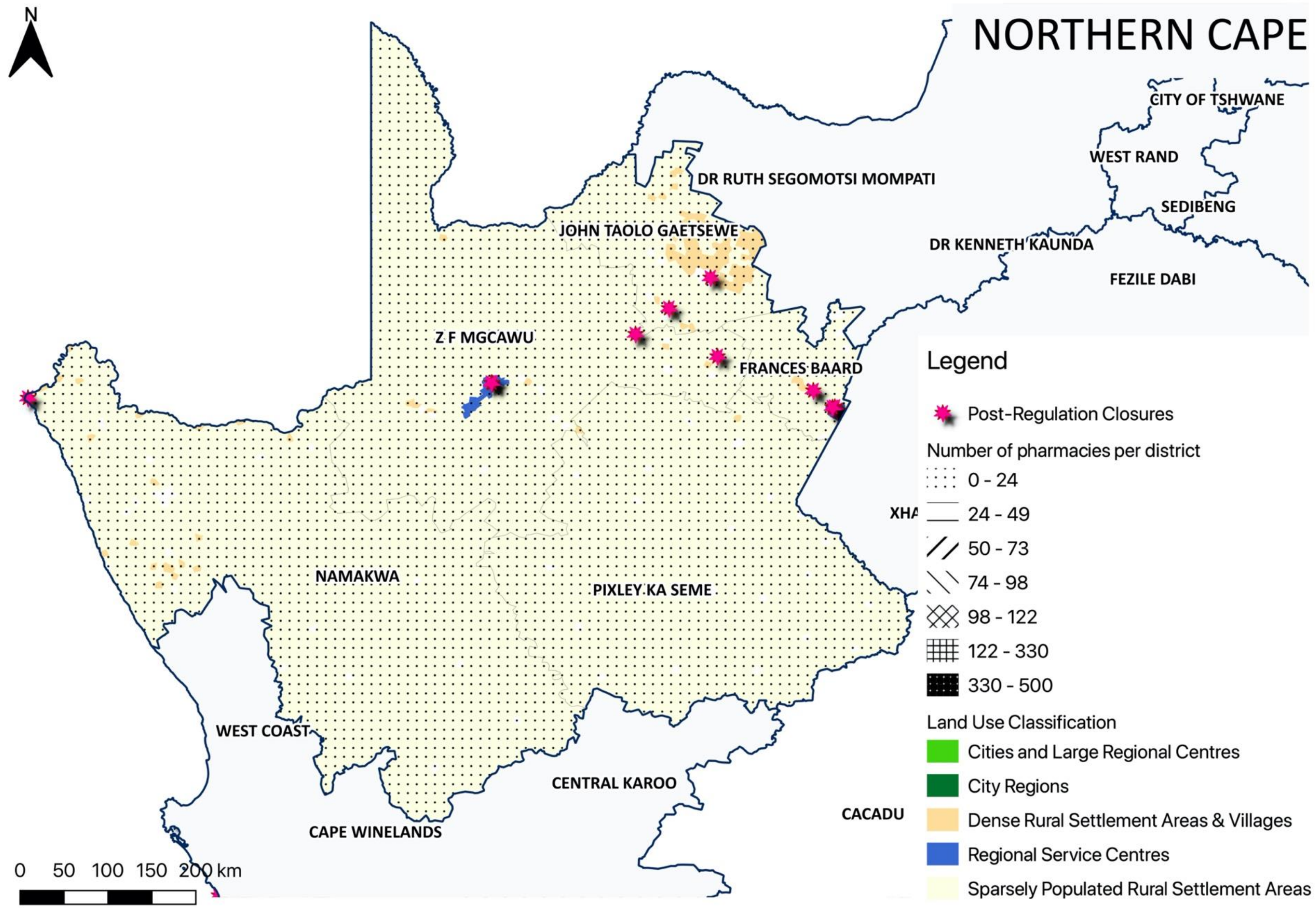

ACTIVE PHARMACIES IN NORTH WEST PROVINCE: REGISTERED PRE-REGULATION

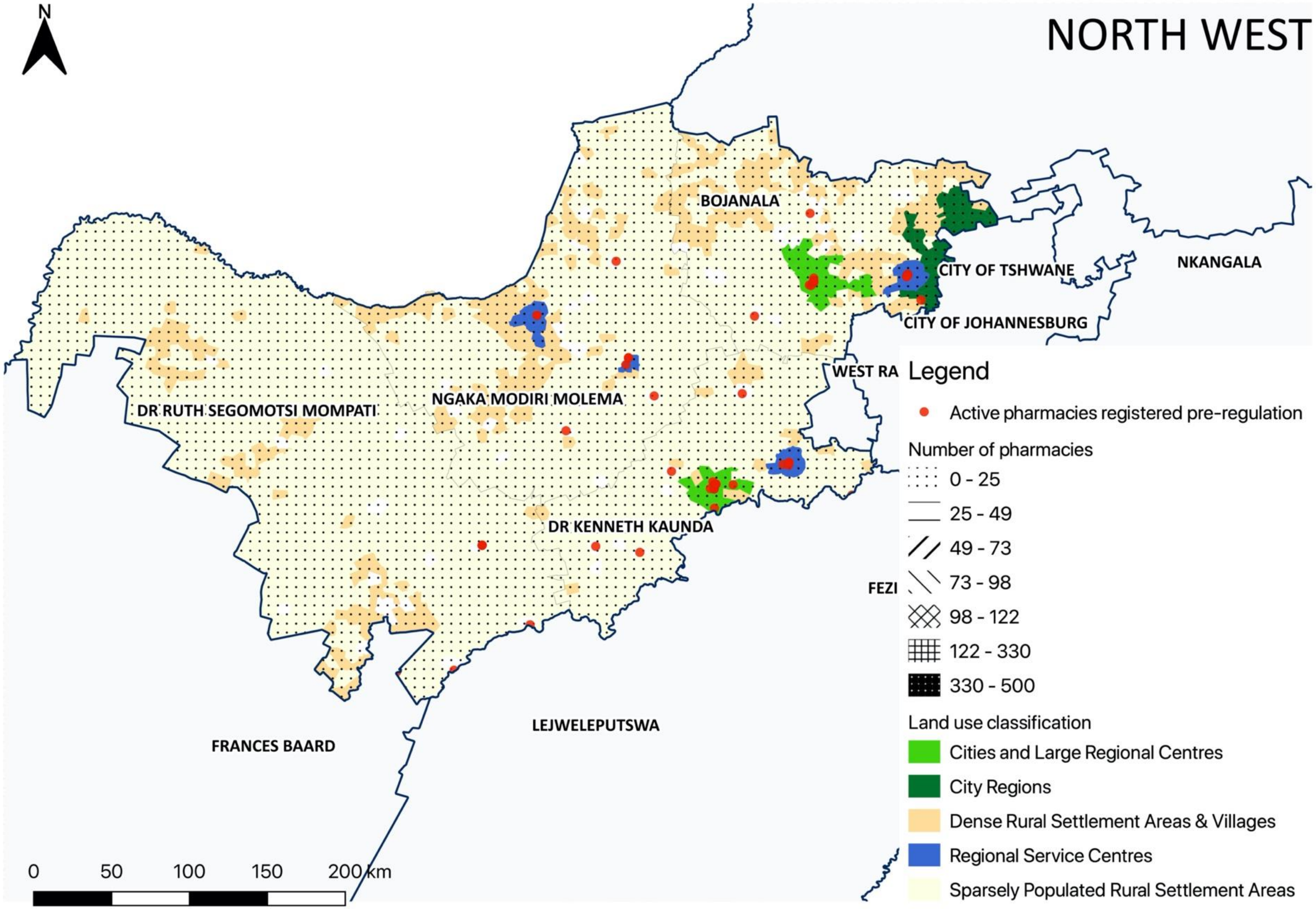

ACTIVE PHARMACIES IN NORTH WEST PROVINCE: PRE-REGULATION VERSUS POST-REGULATION

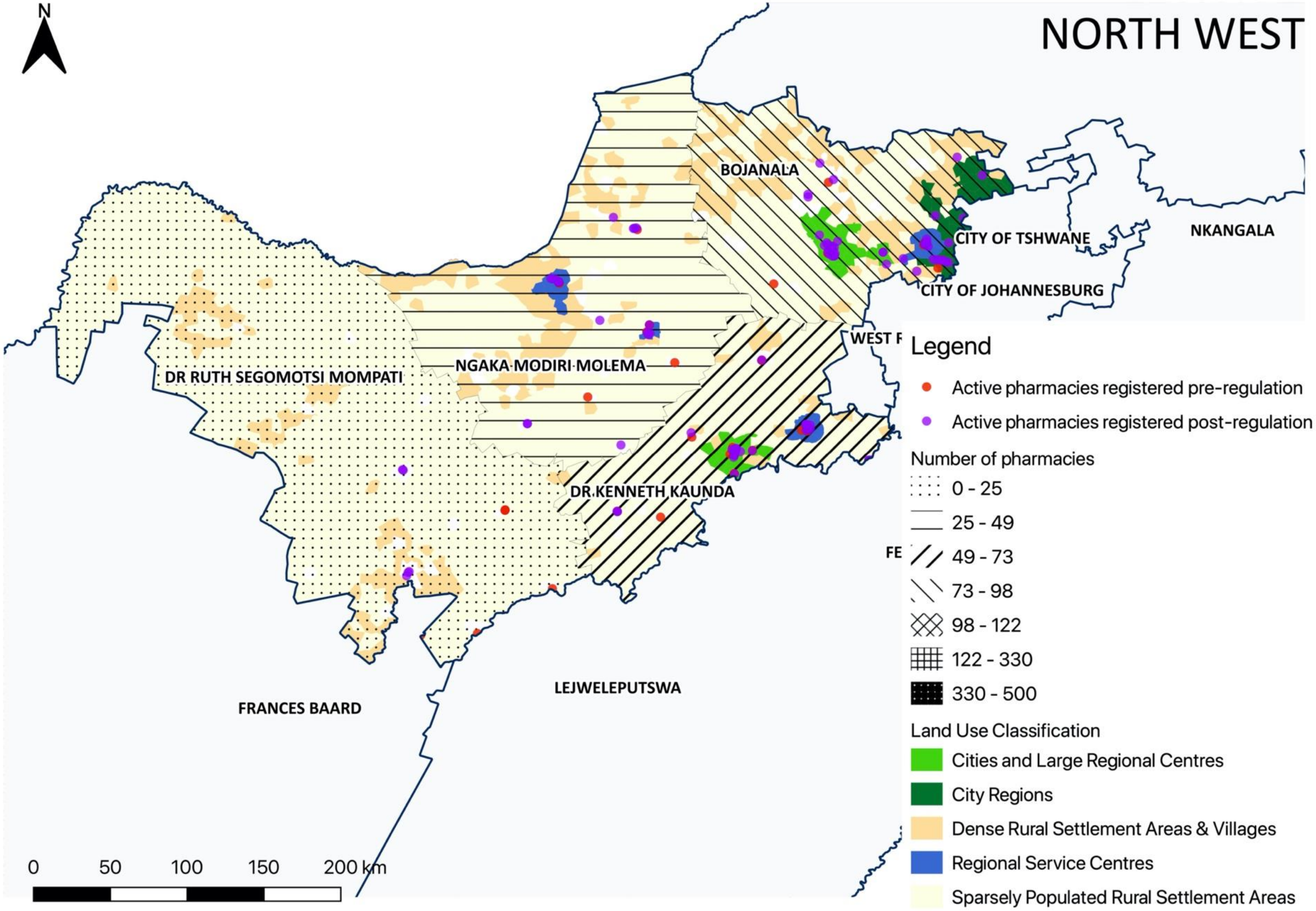

INACTIVE PHARMACIES IN NORTH WEST PROVINCE: POST-REGULATION CLOSURES

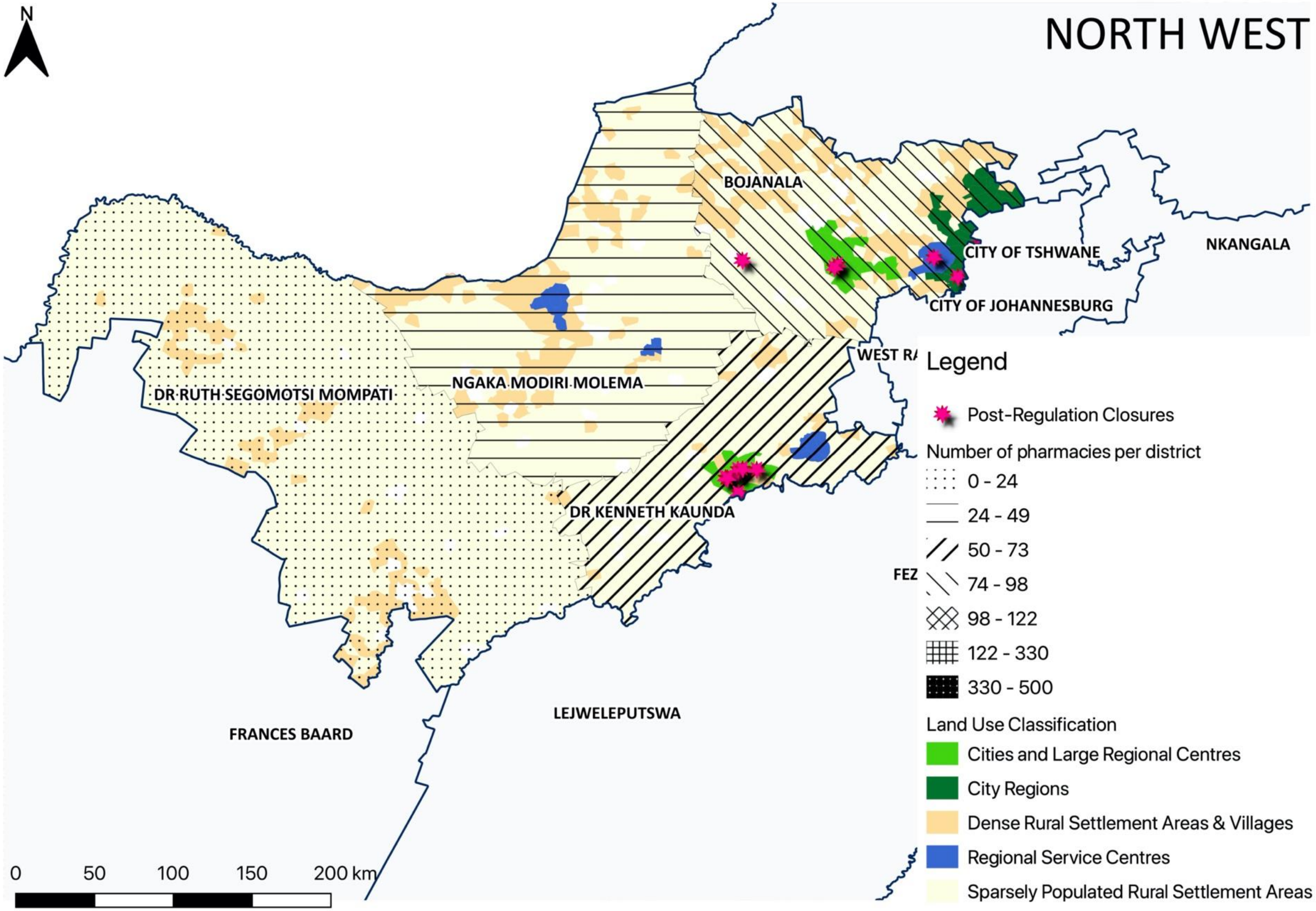

ACTIVE PHARMACIES IN THE WESTERN CAPE: REGISTERED PRE-REGULATION

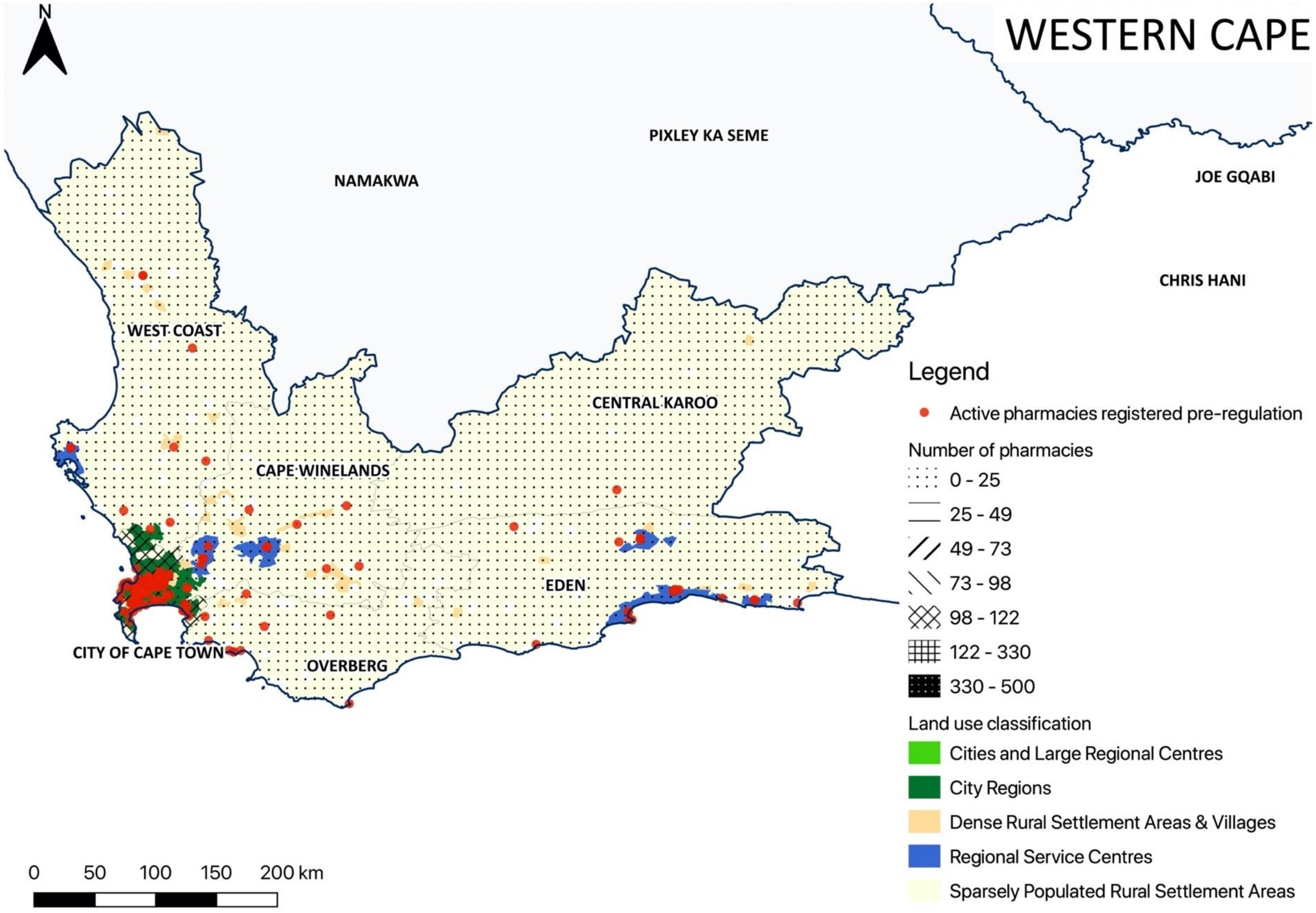

ACTIVE PHARMACIES IN THE WESTERN CAPE: PRE-REGULATION VERSUS POST-REGULATION

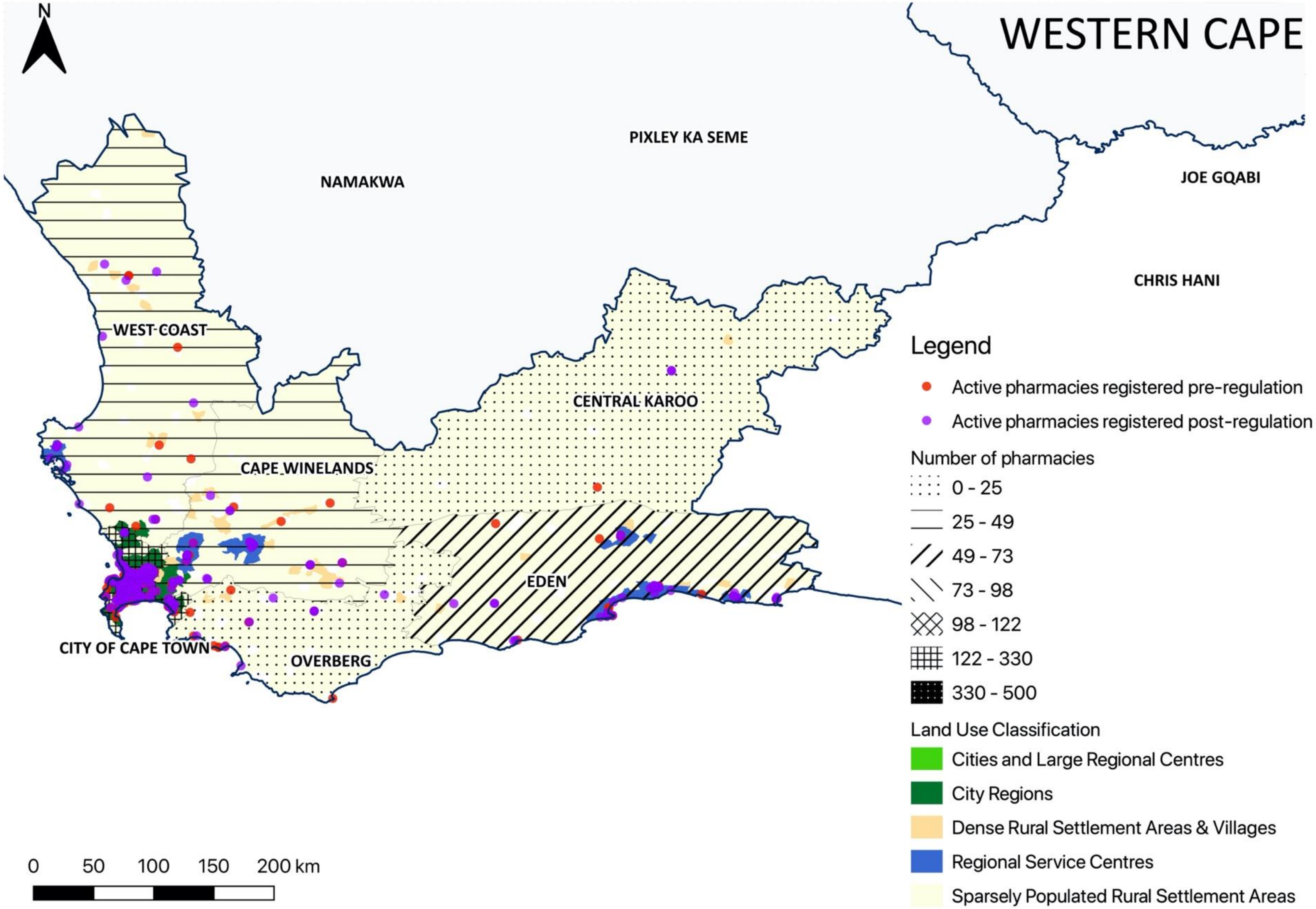

INACTIVE PHARMACIES IN THE WESTERN CAPE: POST-REGULATION CLOSURES

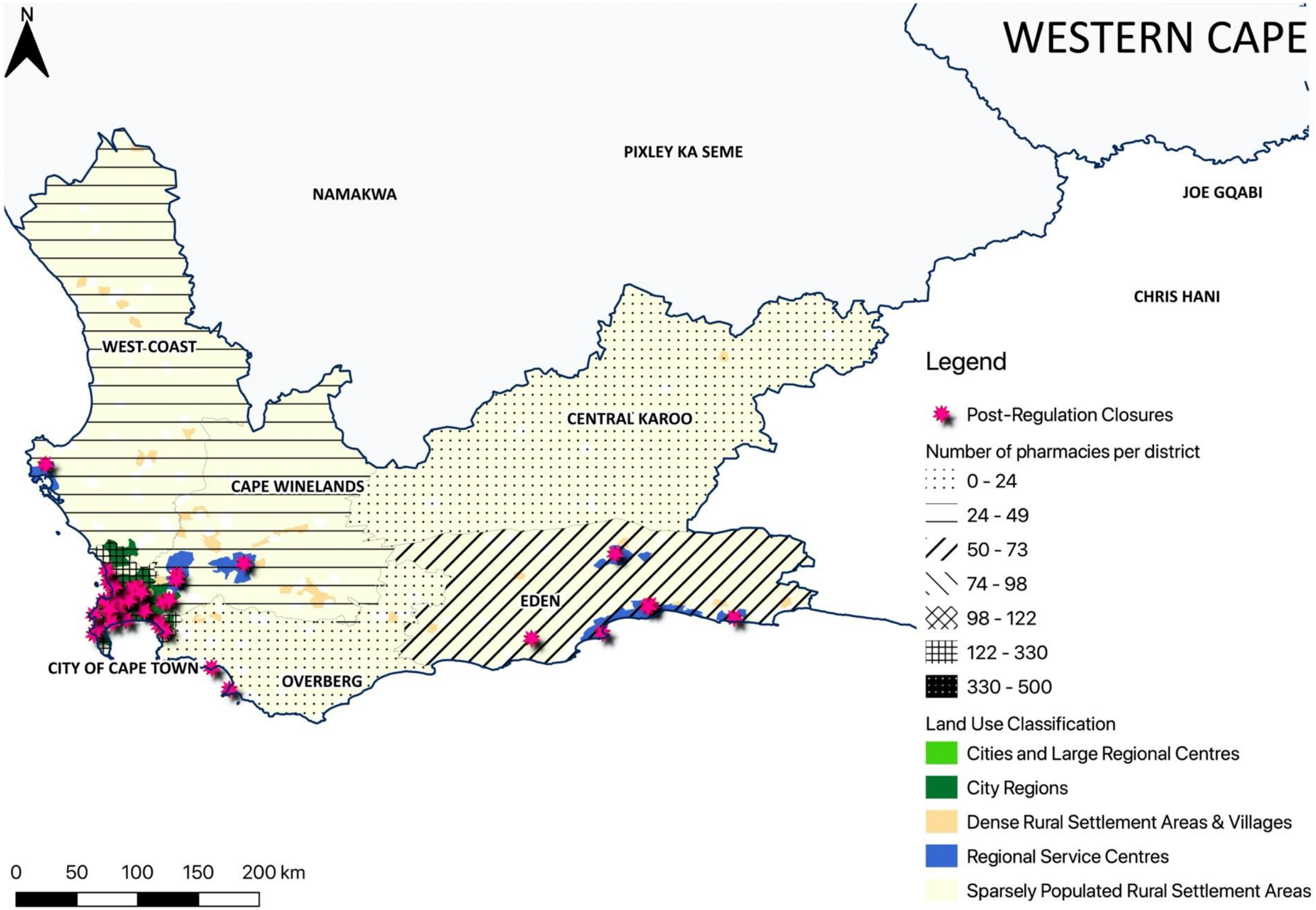

Supplement: Supplementary file 1 — Additional file 1. Active pharmacies in the eastern cape: registered pre-regulation. [file 40545_2020_232_MOESM1_ESM.pdf]
